# Research on intelligent decision support systems for oil and gas exploration based on machine learning

**DOI:** 10.1371/journal.pone.0314108

**Published:** 2024-12-05

**Authors:** Zisong Wang, Zhiliang Cheng, Xiujian Ding, Lu Xia

**Affiliations:** 1 School of Civil Engineering and Transportation, Weifang University, Weifang, Shandong, China; 2 School of Petroleum Engineering, China University of Petroleum (East China), Qingdao, Shandong, China; 3 Shandong Institute of Petroleum and Chemical Technology, Dongying, Shandong, China; SR University, INDIA

## Abstract

The process of extracting oil and gas via borehole drilling is largely dependent on subsurface structures, and thus, well log analysis is a major concern for economic feasibility. Well logs are essential for understanding the geology below the earth’s surface, which allows for the estimation of the available hydrocarbon resources. The incompleteness of these logs, on the other hand, is a major hindrance to downstream analysis success. This study, however, addresses the above challenges and presents a deep Long-Short Term Memory (LSTM) model specialized using a new hyperparameter tuning algorithm. There is an evidence gap that we try to fill: well log prediction using LSTM has not been extensively documented, particularly on reconstruction of missing data. In order to remedy this, we develop a new algorithm entitled Elite Preservation Strategy Chimp Optimization Algorithm (EPSCHOA), which will improve the tuning of LSTM hyperparameters. EPSCHOA enhances prediction performance by preserving the diversity of the strongest candidates and transforming the most effective predictor resources into less effective ones. A comparative analysis of the LSTM-EPSCHOA model was carried out with both LSTM and E-LSTM models, including their various extensions, LSTM-CHOA, LSTM-HGSA, LSTM-IMPA, LSTM-SEB-CHOA, and LSTM-GOLCHOA, even as common forecasting models using Artificial Neural Network (ANN), Adaptive Neuro-Fuzzy Inference System (ANFIS), Gradient Boosting (GB), and AutoRegressive Integrated Moving Average (ARIMA). The results of the performance tests demonstrate that the LSTM-EPSCHOA model outperforms in all aspects, as evidenced by its R^2^ values of.98, RMSE of 0.022, and MAPE of 0.701% during training, and R^2^ values of 0.96, RMSE of 0.025, and MAPE of 0.698% during testing. These are considerably superior to other measures used compared to what was achieved using explicit modeling using LSTM, which stood at R^2^ of 0.59, RMSE of 0.101, and MAPE of 2.588%. The LSTM-EPSCHOA proved to give models faster rates of convergence and lower error measurements than usual models, which clearly demonstrated its efficiency in solving the problem of inadequate well-log data. The new approach is regarded as having many useful potentials to boost well-log interpretations in the oil sector.

## Introduction

The proliferation of machine learning (ML) techniques applied to real-world situations is a direct result of the exponential growth in computing power and the rapid development of ML in recent years [1–3]. Because of its abundance of data, the petroleum industry is in a prime position to reap substantial benefits from these methods [4–6]. Typically, this data is comprised of physical property measurements taken in well logs—serial recordings of features acquired at normal depth increments—during exploratory borehole drilling [7, 8].

In order to infer geological features surrounding a well, a number of modeling programs take the well logs as input. Commercial decisions regarding the wells or an entire oilfield’s development might be informed by these, which are based on the anticipated existence of hydrocarbons [9–11]. Interpreting well logs—which take raw measurements and turn them into commercially viable information—is a labor-intensive and costly process that needs much human expertise [12–14].

Missed chances or the expense of digging an oil well with poor hydrocarbon yield are two ways in which erroneous hydrocarbon content forecast can significantly affect the economy [15, 16]. The well logs are imperfect and prone to noise because they document raw measurements taken by different instruments. So, the well logs are “conditioned,” or cleaned, by correcting inaccurate measurements and approximating missing values. This process requires a significant amount of time and effort from the petroleum engineers responsible for the interpretation [17, 18]. They cannot go on to interpret the rock qualities until this is done [19].

Recent research [20] sought to automate the well-log interpretation process with little human input using ML, with the goal of reducing the time required from seven days of human work to seven minutes. The large amount of missing data in the logs was one of the primary challenges found in that effort. This is due to the fact that the measurements are inputs to the prediction networks, and their extremely infrequent absence significantly limits the quantity of valuable data or choosing of appropriate models. This paper’s work has double advantages: first, petro-physicists will be able to derive interpretations with more certainty if the well log gaps can be estimated with sufficient accuracy; second, a plethora of different ML methods can be unlocked for additional analysis of the well logs.

We employ a hybrid supervised ML method named LSTM-EPSCHOA to determine the connections between several borehole-measured physical characteristics in this study. Consequently, we take advantage of one of these characteristics as a dependent variable and employ the others as independent ones. Every entry in a well log stands for one observation; we use a portion of these observations to train the models while keeping a separate set for evaluation. We exclusively employ data with recorded values of the desired attribute for training and evaluation, with the measured value serving as the ground truth. As a result, petro-physicists will not have to label the data in advance, which, according to our theory, will lead to higher quality data values free of biases induced by humans.

In conclusion, this study primarily aims to present and assess the LSTM-EPSCHOA model for estimating well logs missing values. The paper also uses an empirical dataset to compare the LSTM-EPSCHOA model’s performance to that of traditional LSTM procedures, as well as other well-known machine-learning approaches. The assessment is conducted using many measures.

The following is the paper’s outline: Related works section gives some overview of related works to set the stage for this effort. Related terminology section presents the background on the subject. Proposed methodology section details the proposed model that was employed to forecast the missing data. Experimentation section represents the experimentations and obtained results, and conclusion and recommendations section provides a summary of our discoveries.

## Related works

Recently ML, fuzzy system and deep learning techniques have been proposed to solve the complicated engineering problems [21–24]. Engineers rely significantly on their knowledge and expertise of supplementary, non-logged data for hydrocarbon finding and extraction modeling and interpretation. They are not ideal candidates for automated computational methods, however, items like drilling records and images of borehole waste are part of these considerations [25]. A set of sufficiently large well logs may, however, implicitly incorporate the experience of petro-physicists, according to a hypothesis. In this part, we provide a summary of the notable work that has investigated the use of ML approaches to related challenges.

Seismic measurements are investigated by Liu and Sacchi [26] as a means of transmitting data regarding physical rock qualities from existing (full) well logs to future (unfinished) ones. Although the results show promise, seismic readings are currently applied to evaluate rock models that are obtained from well logs instead of input in petrophysical procedures. Our decision to leave out the seismic measurements was based on the fact that using them earlier in the interpretive workflow would make this kind of validation impossible.

The issue of lost information in well logs is tackled by Holmes et al. [27] using ML algorithms that are based entirely on the rock physics measurements that are available. In addition to outlining potential problems, the document also includes steps to fix them. One of their main points is that because geology varies over intervals, the typical method of using neural networks—namely, training the network on existing information and then predicting the lost data—may not be applicable to well logs. They recommend manually preprocessing the data and using proven procedures to make sure the machine does not learn incorrect inferences. Instead, they suggest intervals that share comparable geological characteristics for training. In addition, the authors suggest that specialists use neural networks in conjunction with predictable petrophysical and stochastic mathematical models to determine which of the three forecasts is the most feasible. Because a person has to be involved, this method is not as accurate or reliable, and it cannot handle a lot of well logs. It also adds a bias.

In order to forecast the missing values, Lopes and Jorge [28] employed various ML models trained on eight well logs; however, they failed to account for the possibility that the logged data might contain issues due to geologically erratic behaviors. After doing an in-depth descriptive and exploratory examination of the gaps, the authors of the research assess the effects of linear models, Gradient Tree Boosting, Random Forests, and Artificial Neural Networks on n prediction. We build upon their previous work by utilizing a more extensive dataset and assess the models across four distinct prediction objectives. Additionally, we look into how the quantity and closeness of the training data affect the accuracy of models.

Using RNN—more significantly, a CLSTM (cascaded)—Zhang et al. [29] demonstrated a method for completing the missing measurements. According to the authors, the outcomes are more precise than what a conventional Neural Network would predict. While it is true that LSTMs make better use of spatial dependencies in well logs, the results shown here are based on a single well log, and previous research has shown that Training a model that can accurately predict outcomes for a large number of wells is considerably more challenging [30]. The ability to finish just one log makes it hard to compare methods since we have found that particular well logs are simpler to produce than others.

To facilitate better understanding of our approach, Table 1 below compares several previous works, portraying the key points of difference and performance enhancement that can be brought to this enumeration style.

**Table 1:** Detailed breakdown of studies related to missing data in automation of processes in well logging.

References	Models	Geological Variability	Dataset Size	Limitations	Our Novelty	RMSE (Performance)
Liu and Sacchi [26]	Petrophysical models, seismic measurements,	Not addressed	Small	Not applicable in petrophysical workflows, Limited to seismic data	Excludes seismic measurements for interpretive consistency	N/A
Holmes et al. [27]	Stochastic models, ANN	Handled manually	Moderate	Introduces bias, Requires manual preprocessing	Fully automated using LSTM and EPSCHOA for missing data estimation	0.036
Lopes and Jorge [28]	Random forest, Neural networks, Gradient tree boosting	Not addressed	Moderate	Ignores geological variability	Utilizes EPSCHOA for adaptive and dynamic hyperparameter tuning	0.032
Zhang et al. [29]	Cascaded LSTM (CLSTM)	Not addressed	Small (single well)	Focused on a single well log	Applies LSTM-EPSCHOA to multiple wells for more generalizable results	0.029
**This Study**	LSTM with EPSCHOA	Handled via model tuning	Large (NLOG)	None	Novel EPSCHOA for hyperparameter optimization and superior missing data estimation	**0.024**

Explanation of table and novelty:

- *Handling of Geological Variability*: Unlike previous works, our model uses EPSCHOA to seasonally modify LSTM hyperparameters in real time, making it more effective at handling geological variability in the data, a feature that other studies rarely capture.
- *Automation and Scalability*: EPSCHOA integration ensures full design optimization, automating preparations that Holmes et al.’s study [27] only partially performs manually.
- *Generalizability*: Unlike the rest, Zhang et al. [29] limited themselves to testing their approach on a single well log. They test their approach on a larger dataset (NLOG) and demonstrate its superior prediction generalization for multiple wells.
- *Performance*: As shown in the table, our proposed LSTM-EPSCHOA model yields the least RMSE compared to other models and thus gives the best prediction of the missing well log data.

To sum up, the research gaps, motivations, and primary contributions can be listed as follows:

### Primary contributions

A number of factors contributed to the final selection of CHOA over competing, recently introduced optimization algorithms that drew inspiration from nature or used swarm intelligence [31]. The simplicity of its mathematical structure, the limited number of parameters that may be adjusted, and its ability to solve various optimization problems are some of these factors [31, 32].

However, when trying to solve complicated issues, a typical problem that frequently occurs is the capacity to get stuck in the local optimal situation [33–35]. Thus, in order to enhance CHOA’s performance when faced with complex optimization problems, this paper presents the novel EPS technique.

A brief overview of the paper’s main contributions is as follows:

- The paper delves into the use of a LSTM to enhance the estimation of well logs’ missing values. The capacity of LSTM to adequately capture complicated and dynamic connections within well-log data is an essential consideration in its selection.
- The EPSCHOA is an innovative technique for hyperparameter tuning that is developed in this paper. The difficulty of fine-tuning LSTM’s hyperparameters is the inspiration for this algorithm.
- EPSCHOA promotes variety among high-performing individuals while simultaneously boosting the performance of searching agents with less valuable performance, thus enhancing the search procedure within the optimization algorithm. The goal of this strategy is to explore the search space efficiently.
- This study evaluates the performance of various versions of the LSTM, including typical LSTM, as well as variants modified with optimization algorithms like LSTM-HGSA, LSTM-IMPA, and LSTM-GOLCHOA.
- The research assesses how well various LSTM variations perform on NLOG, a real-world dataset. In this assessment, bias metrics, NSEF, and root-mean-squared error (RMSE) are utilized.
- Using the measures mentioned above, the study found that the suggested LSTM-EPSCHOA prediction model performed better than other variations. The model’s estimating well logs missing data with outstanding performance is suggested by this model.

To summarize, the main contributions of the paper are the EPSCHOA algorithm for LSTM hyperparameter tuning and the comprehensive evaluation of different LSTM versions for well-log missing values estimation. These enhancements demonstrate the efficiency and potential of the algorithms for real-world missing value estimation.

## Related terminology

This section reviews the necessary background information for well logging, CHOA, and LSTM.

### Well logging

**Fig 1:**
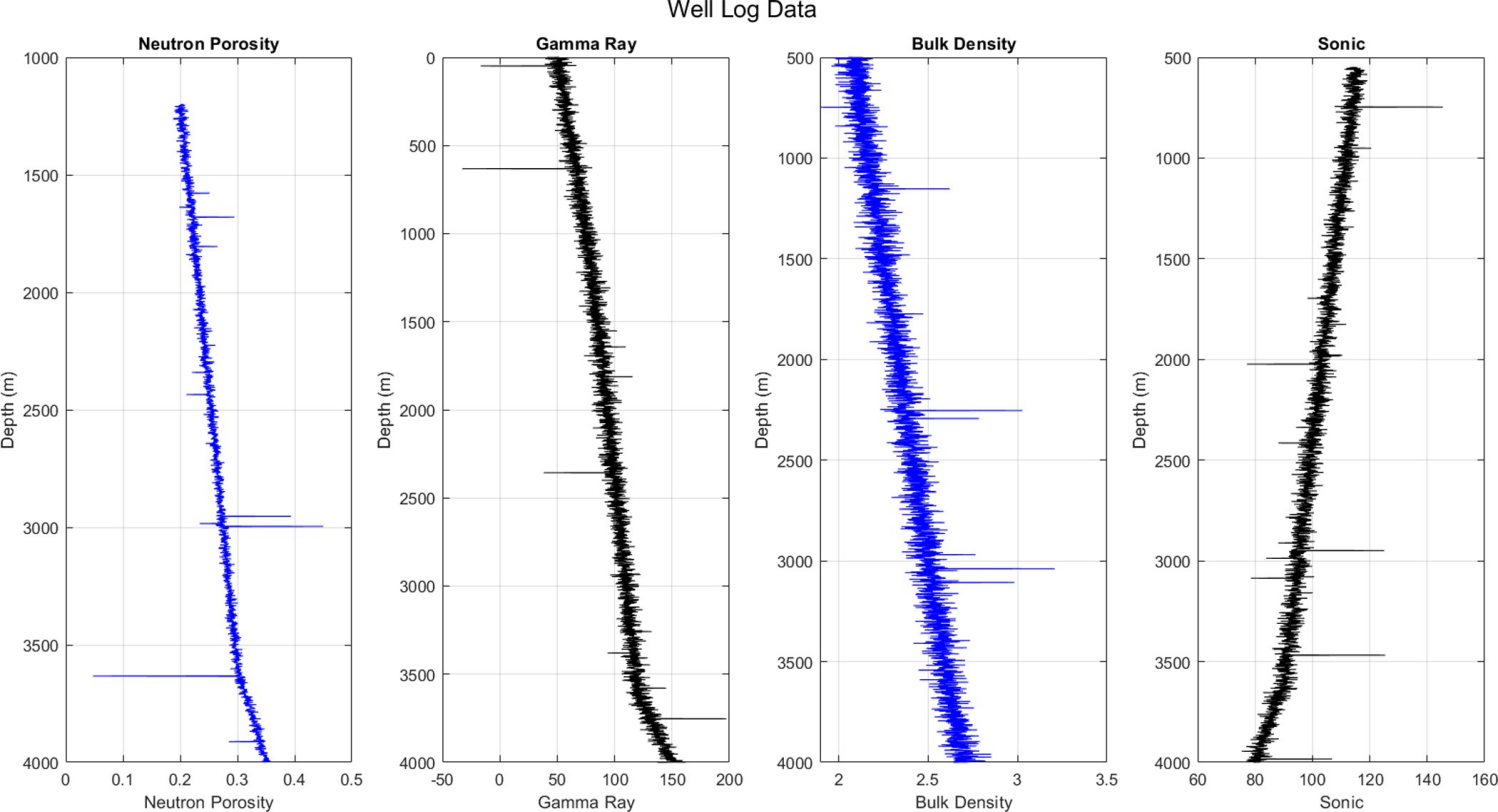
Typical vertical graph that displays collected values of these qualities side by side.

Well logging [36, 37] is a technique used in the oil and gas sector to record data from the rock formations that have been passed by lowering measurement instruments called sounds down a borehole at regular intervals. In order to ascertain, among other things, the anticipated hydrocarbon yield, petro-physicists painstakingly examine this raw data. Fig 1 shows a typical vertical graph that displays collected values of these qualities side by side, where x-axis represents the characteristic and y-axis represents depth. This allows petro-physicists to mentally see the borehole and its features, which helps them infer the mineralogy and hydrocarbon content at different depths. We focus on the most frequently observed rock properties—gamma ray, neutron porosity, bulk density, and sonic—in our work so that most of the well logs can be used.

It is a unitless number that describes how porous the rock is and how much fluid is in the pores. This makes neutron porosity (*n*_*p*_) a secondary way to find out how much hydrocarbon is inside. The Gamma-ray (*g*_*r*_) score provides insight into the mineralogical makeup of the local geology by measuring the natural radiation that is emitted by the rocks in the area. The absorption of gamma rays that are intentionally created gives rise to the bulk density (*b*_*d*_), which is a measure of the rock’s consistency. Sonic measurements (*s*_*m*_) quantify the amount of time it takes for a sound wave to travel a certain distance within a rock formation, providing further information about the lithology and porosity. Because the rock’s permeability and the type of fluid that flows through its tiny openings, this provides valuable information.

Regular depth intervals are used to measure and record the values of various attributes. However, there is a challenge in that some levels may have missing values. Mistakes made by humans or problems with the sondes are two of the many potential causes of missing data. At some levels, even financial constraints could cause measurements to be withheld because they are deemed too costly to be practical to collect.

### Chimp optimization algorithm

The CHOA is an optimization technique developed by studying chimpanzee behavior, namely their eating patterns. When CHOA goes on a hunt for food, it mimics the strategies used by chimpanzees in the wild [38–40].

The CHOA is a method for optimizing processes that was inspired by research into chimpanzee behavior, including their feeding habits [41, 42]. In its search for food, CHOA acts similarly to how wild chimpanzees do [43–45]. The approach keeps an eye on a population of solutions to the optimization problem until it identifies one that could work. By fusing the two phases of the search process, CHOA imitates the way chimpanzees find their way around [46–51]. By taking a variety of perspectives into account, CHOA promotes a wide range of potential solutions throughout the study phase. This action is similar to how chimpanzees search for food in new environments. While in the exploitation phase, CHOA explores a constrained area of the solution space exhaustively in an effort to uncover realistic solutions. This is similar to how chimpanzees gorge themselves on food they have already found [32].

The capacity to dynamically adjust exploration and exploitation rates in response to population changes is one of several ways CHOA strikes a balance between the two. The CHOA framework includes techniques for avoiding early convergence as well as ways for managing restrictions and preserving population diversity [31].


$$L_{chimp}^{t+1}=L_{\text{prey}}^{t}-\kappa \cdot |J\cdot L_{\text{prey}}^{t}-\zeta \cdot L_{chimp}^{t}|$$ (1)

$$\kappa =2\cdot Q\cdot r_{1}-Q$$ (2)

$$J=2\cdot \left(r_{2}\right)$$ (3)

ζ=chaoticmaps (4)

**Fig 2:**
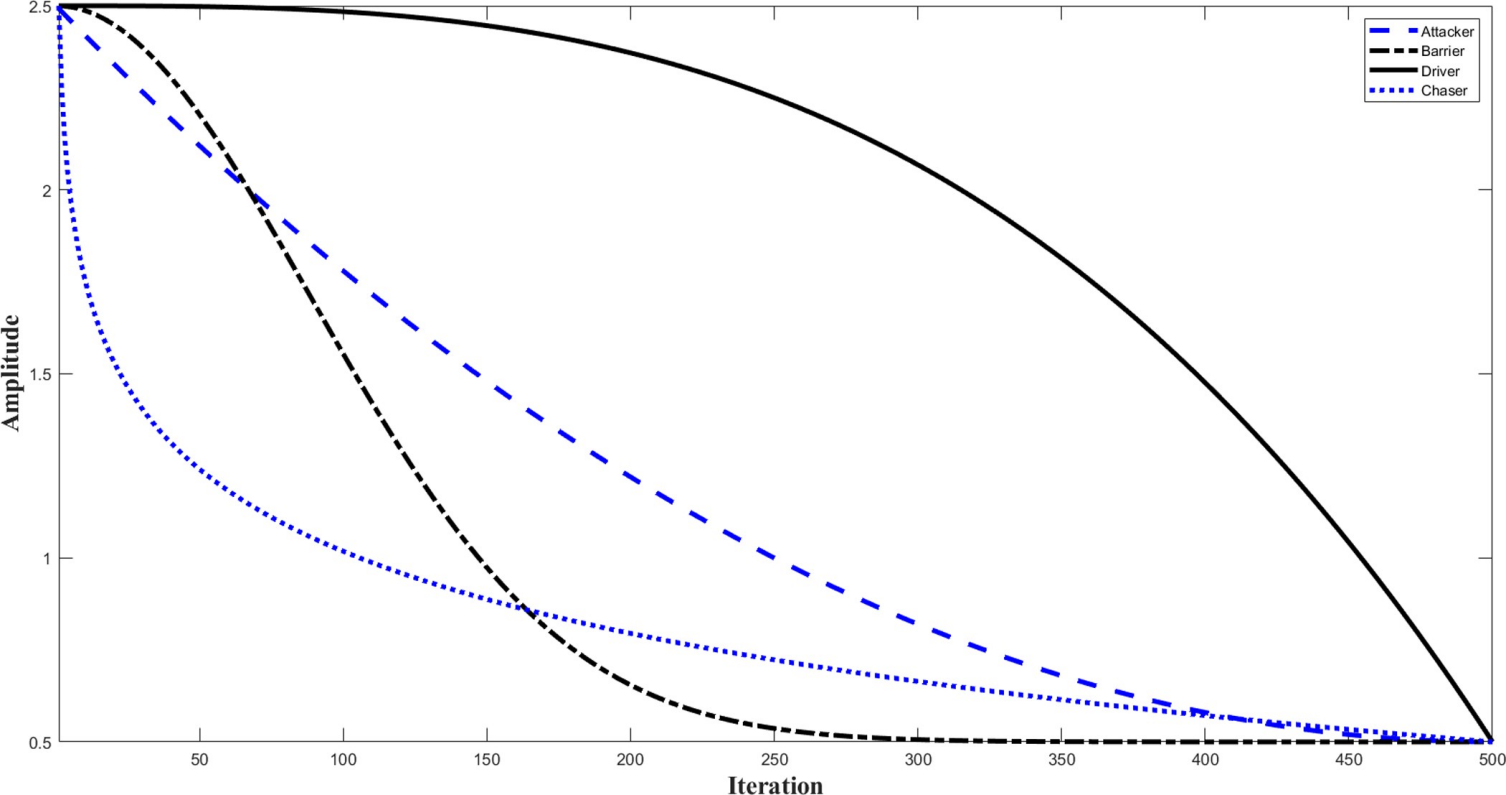
Mathematical presentation of attacker, barrier, chaser, and deriver’s coefficients.

**Fig 3:**
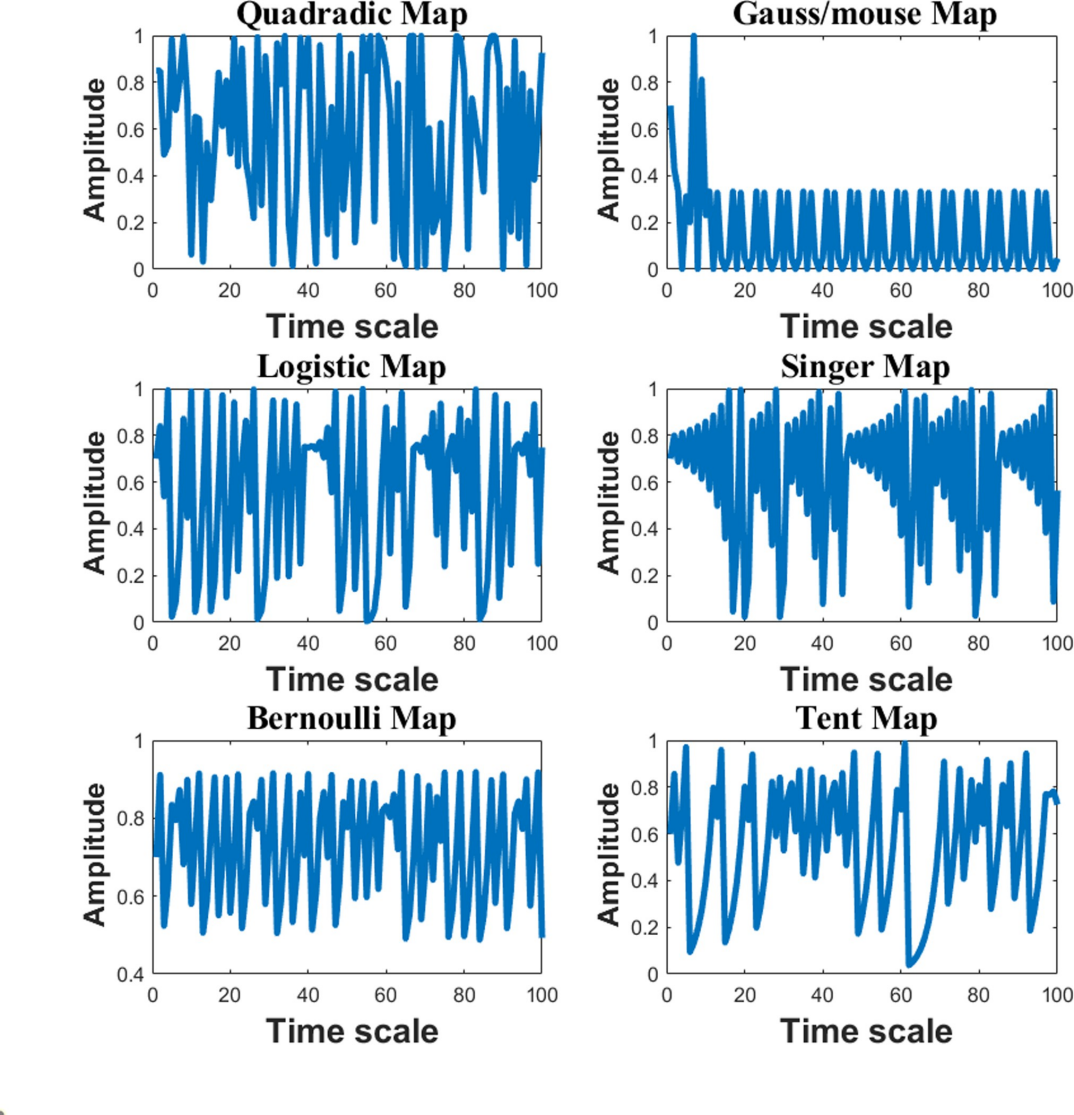
Chaotic vectors.

The ideal chimpanzee environment is represented by *L*_prey_, the optimum solution is denoted by *L*_*chimp*_, and the entire number of iterations is represented by *t*. As illustrated in Fig 2, there is also a non-linear *Q* coefficient whose value falls somewhere between 2.5 and 0. Within the range of 0 to 1, two integers, **r**_**1**_ and **r**_**2**_, are totally arbitrary. Fig 3 shows the vectors of the chaos map. Remember that the reference gives a more detailed study of these maps and coefficients [31].

Due to the lack of information about where the first food came from, the most skilled and early chimpanzees learned to manipulate their prey in the same way that chimpanzees did. After CHOA has taken up residence with the best four chimps so far, the other slots will be filled according to the criteria laid down by Eqs (5) and (6) [31].


$$L^{t+1}=\frac{1}{4}\times (L_{1}+L_{2}+L_{3}+L_{4})$$ (5)

Where

$$\begin{array}{l} L_{1}=L_{A}-a_{1}\cdot \left|c_{1}L_{A}-m_{1}L\right|\;\;\\ L_{2}=L_{B}-a_{2}\cdot \left|c_{2}L_{B}-m_{2}L\right|\\ L_{3}=L_{C}-a_{3}\cdot \left|c_{3}L_{C}-m_{3}L\right|\\ L_{4}=L_{D}-a_{4}\cdot \left|c_{4}L_{D}-m_{4}L\right| \end{array}$$ (6)

Eq (7) demonstrates that classic CHOA’s chaotic values behave similarly to social incentives.


$$L^{t+1}=\left\{\begin{array}{l} \frac{1}{4}\times (L_{1}+L_{2}+L_{3}+L_{4})\;\;\eta_{m}<0.5\\ \zeta \;\eta_{m}\geq 0.5 \end{array}\right.$$ (7)

The value *η*_*m*_ is chosen at random from the interval [0,1].

## Proposed methodology

In this section, we first present the peculiarity of the proposed method. It is suggested that an EPSCHOA algorithm be developed then, followed by the SLMS model, and lastly, that the model be optimized using the EPSCHOA method.

### Distinctiveness of the proposed methodology

Here, the proposed LSTM-EPSCHOA framework incorporates several novel and innovative components that distinguish the theory from previous approaches to well log data analysis and missing value filling. Such distinguishing factors include:

- **Chimp optimization algorithm with elite** **preservation strategy (EPSCHOA)**

This work is unique in that it presents the EPSCHOA algorithm, which optimizes the hyperparameters of LSTM models in a novel manner. EPSCHOA implements an elite preservation method that retains the top-performing optimization candidates for further search improvements, while simultaneously enhancing the weaker candidates. The model thus balances between exploration and exploitation; at no point does it reach local optima, a problem with most optimization aiming for complex well log datasets.

- **Dynamic** **hyperparameter tuning**

In contrast to traditional tuning, which uses pre-determined static hyperparameters, the proposed methodology uses EPSCHOA for hyperparametric tool adjustment. Therefore, it is possible to make effective changes to such hyperparameters (learning rate, batch size, or number of LSTM layers) anytime during the training. Such flexibility helps in reducing the extent to which an LSTM model becomes overly specialized or ‘overfitted’ to the particular geological features present in the well log data.

- **Efficient** **handling of missing data in well logs**

Thanks to the most timeseries form of data in LSTM, such deep learning technology is able to address the issue of missing data in well logs. The non-stationary nature of well log data causes historical models like ANNs and gradient boosting to perform poorly, while the LSTM-EPSCHOA approach effectively encodes both the short-term and long-term dependencies of the data, resulting in a more spherical picture and reducing the underestimation of missing values. This flexibility in dealing with complex time-ordered data is what separates this from most machine learning models.

- **Scalability across** **large and complex datasets**

Previous works have only focused on small datasets or single-well logs. This work aims to enhance the LSTM-EPSCHOA framework by offering a scalable solution for the large NLOG dataset, which includes a variety of well logs with incomplete information. The scalability of the model enables top-notch industrialized deployment, which is beneficial for the oil and gas industries.

- **Better performance** **compared to other algorithms**

To assess and compare its performance, several models are used against the LSTM-EPSCHOA model, including the LSTM-HGSA, LSTM-IMPA, and LSTM-GOLCHOA. The proposed model performs more accurately when reconstructing missing log data using the RMSE, R2, and MAPE metrics. The EPSCHOA had a net gain because the sequential aspects improved the model hyperparameter tuning of the LSTM, resulting in better predictions.

- **Low algorithm** **complexity and low resource usage**

EPSCHOA aims at enhancements of the search in terms of efficiency without too many additional computations. In this respect, the LSTM-EPSCHOA framework appropriately ignores other non-promising search agents and concentrates on a small number of better candidates, resulting in better performance at less computational costs when compared with other optimization strategies. This, however, improves the cost efficiency of the method and makes it applicable in time-limited activities.

### Elite preservation strategy chimp optimization algorithm

Using a unique approach, Saremi et al. [52] demonstrated a way to find and remove 50% of the population’s least effective search agents. The underperforming agents are placed in close proximity to the agents who have produced exceptional results, as determined by their objective values when they are re-acquired. Instead of concentrating on the best answers, this approach promotes coming up with replacements with lower objective values. We can fairly distribute the least influential persons among four chosen at random places by using Eqs (8)–(12).


$$\begin{array}{l} L^{t+1}=L_{A}^{t}\pm (L_{b}+z_{1}(U_{b}-L_{b}))\\ \text{if}\;0\leq z_{6}\leq 0.2 \end{array}$$ (8)

$$\begin{array}{l} L^{t+1}=L_{B}^{t}\pm (L_{b}+z_{2}(U_{b}-L_{b}))\\ \text{if}\;0.2\leq z_{6}<0.4 \end{array}$$ (9)

$$\begin{array}{l} L^{t+1}=L_{C}^{t}\pm (L_{b}+z_{3}(U_{b}-L_{b}))\\ \text{if}\;0.4\leq z_{6}<0.6 \end{array}$$ (10)

$$\begin{array}{l} L^{t+1}=L_{D}^{t}\pm (L_{b}+z_{4}(U_{b}-L_{b}))\\ \text{if}\;0.6\leq z_{6}<0.8 \end{array}$$ (11)

$$\begin{array}{l} L^{t+1}=L^{t}\pm (L_{b}+z_{5}(U_{b}-L_{b}))\\ \text{if}\;0.8\leq z_{6}\leq 1 \end{array}$$ (12)

The procedure for improving the performance of the poorest solutions to that of the best-performing ones is laid forth in Eqs (8) through (11). Within the scope of the inquiry, Eq (12) centers on the stochastic displacement of the least efficient solutions. Assailant, barrier, pursuer, and driver positions are represented by the starting points of the variables $L_{A}^{t},\;L_{B}^{t},\;L_{C}^{t},\;\text{and}\;L_{D}^{t}$ in the equations given above. The higher boundary vector of the search space is represented by **U**_*b*_ and the lower boundary vector is represented by **L**_*b*_. Also, the vectors *z*_1_–*z*_6_ are produced arbitrarily and distributed uniformly over the range [0, 1]. By solving such an equation, we can redirect resources from ineffective responses to more productive ones. This approach makes the research process more manageable and reduces the number of missed alternatives as the algorithm gets closer to the best results.

### Tuning system

The EPSCHOA approach was developed to exclude individuals with lower objective values, in contrast to systems that only aim to raise the most qualified candidates. The CHOA framework ranks the four agents—assailant, barrier, chaser, and driver—from weakest to most vigorous. These agents are restructured using the method that was described before. However, one potential issue with this strategy is that it may converge too quickly. Individuals with lower targets need to be relocated to areas of the search field with higher objective values for this convergence to be achieved. These individuals may perform better in a more diversified search area. In order to avoid early convergence and progress research, diversity maintenance must be prioritized, particularly when assessing the least effective options [53].

The EPSCHOA framework verifies that the four best solutions from each iteration converge to ensure that the search space is evenly distributed with respect to the best answers. When implemented across the whole search space, the method mentioned above ensures that solution distribution and recovery occur in regions with high target values. Therefore, perfect solutions tend to attract effective solutions, while less successful solutions tend to seek out locations with more significant variance. This calculated maneuver converges on the best answer to the optimization problem by maximizing CHOA’s exploration and exploitation capabilities.

Half of the population, consisting of those with the most significant objective value, is first excluded via EPSCHOA’s selection technique before the remaining population is considered. Having these agents spread out throughout all four solutions demonstrates how diverse the solution space is. It should be noted that the attacker agent is still kept in the memory of the algorithm and is still directing the search agents. On the other hand, adding these search agents to the current population will also put them among the genuinely unique agents. The degree of enthusiasm for solutions can be determined by using Eq (13).


$$\begin{array}{l} EX_{ind}=\underset{j}{\min}\left|Obj^{i}(L)-Obj^{j}(L)\right|\\ j\in \{1,\ldots ,i+1,\ldots ,N\}\\ i=1,2,\ldots ,N \end{array}$$ (13)

The objective values for the *i*th and *j*th solutions are *Obj*^*i*^(*L*) and *Obj*^*i*^(*L*), respectively, where *EX*_*ind*_ is the enthusiasm index of the solution. The top four $L_{A^{ENTH}}^{t},\;L_{B^{ENTH}}^{t},\;L_{C^{ENTH}}^{t},\;\text{and}\;L_{D^{ENTH}}^{t}$ prospects in terms of enthusiasm, are used to direct half of the top agents through the search area. These motions are executed with a 50% probability using Eqs (14)–(17).


$$\begin{array}{l} L^{t+1}=L_{A^{ENTH}}^{t}\pm (L_{b}+z_{1}(U_{b}-L_{b}))\\ \text{if}\;0\leq z_{5}\leq 0.25 \end{array}$$ 14

$$\begin{array}{l} L^{t+1}=L_{B^{ENTH}}^{t}\pm (L_{b}+z_{2}(U_{b}-L_{b}))\\ \text{if}\;0.25\leq z_{5}<0.5 \end{array}$$ (15)

$$\begin{array}{l} L^{t+1}=L_{C^{ENTH}}^{t}\pm (L_{b}+z_{3}(U_{b}-L_{b}))\\ \text{if}\;0.5\leq z_{5}<0.75 \end{array}$$ (16)

$$\begin{array}{l} L^{t+1}=L_{D^{ENTH}}^{t}\pm (L_{b}+z_{4}(U_{b}-L_{b}))\\ \text{if}\;0.75\leq z_{5}\leq 1 \end{array}$$ (17)

By using Eqs (14)–(17), we may find the updated solutions in the first part of the best-fitting solutions. We can maintain a diverse set of answers while maintaining a consistent distribution of probabilities by randomly selecting an equation from a set for each solution. An arbitrary re-initialization is unnecessary because the EPSCHOA successfully preserves variety even in the absence of factor inclusion.

**Fig 4:**
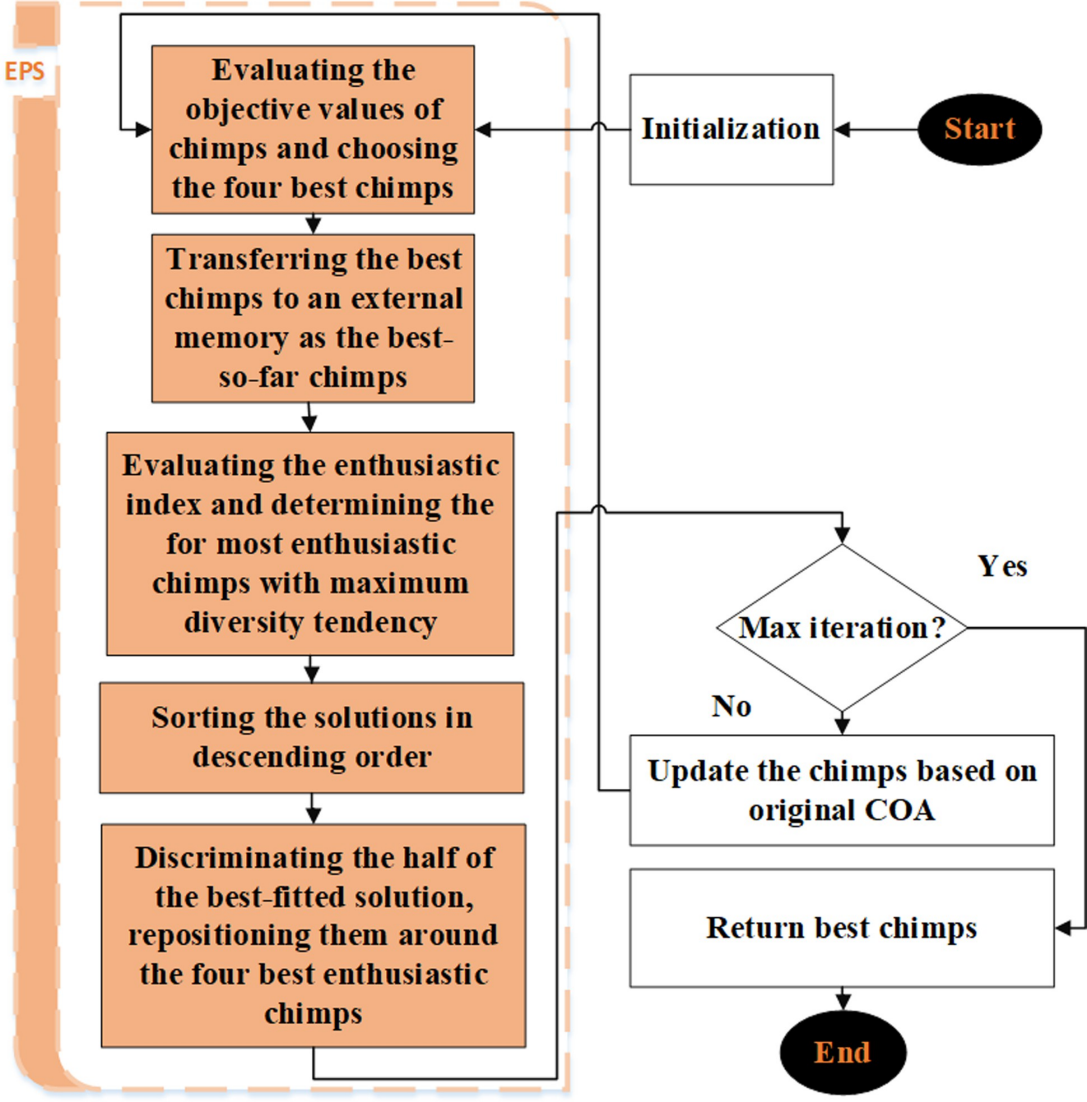
EPSCHOA block diagram.

Fig 4 is a schematic of the method that was created.

### Dataset

The primary data set utilized in this study is the NLOG dataset (https://www.nlog.nl/en), which contains well logs collected in the North Sea shelf area occupied by the Netherlands and is accessible to the public. Due to the exclusive nature of the extra data and its commercial importance to surveying service providers, the well logs that are provided do not include any human interpretation and contain raw measurements.

In accordance with LAS standard [54], the well logs are saved as plain text files, with the log’s general information contained in the file’s header. Included in this set of data are attributes, including the well’s name and coordinates, the starting and ending depths of the borehole, lists of measured properties and the units used to record them5, and the value that stands in for missing measurements (nulls). Next, the file is structured such that each row represents a different well depth, and each tab-separated column stores the value of a single measured attribute. Regular depth increments are used to record the data; the exact step size is mentioned in the LAS file header; however, it usually falls somewhere between 10 and 15 cm.

Although not all rows include these values, bulk density (*b*_*d*_), Gamma-ray (*g*_*r*_), neutron porosity (*n*_*p*_), and sonic measurements (*s*_*m*_) are the parameters that are often documented in the well logs that are currently available. The well logs become incomplete due to these missing features, reducing the amount of information accessible and raising questions about the geological parameters in the area. As an example, out of almost 23,000,000 rows of measurements in the NLOG dataset, only 26,000 rows are complete.

**Fig 5:**
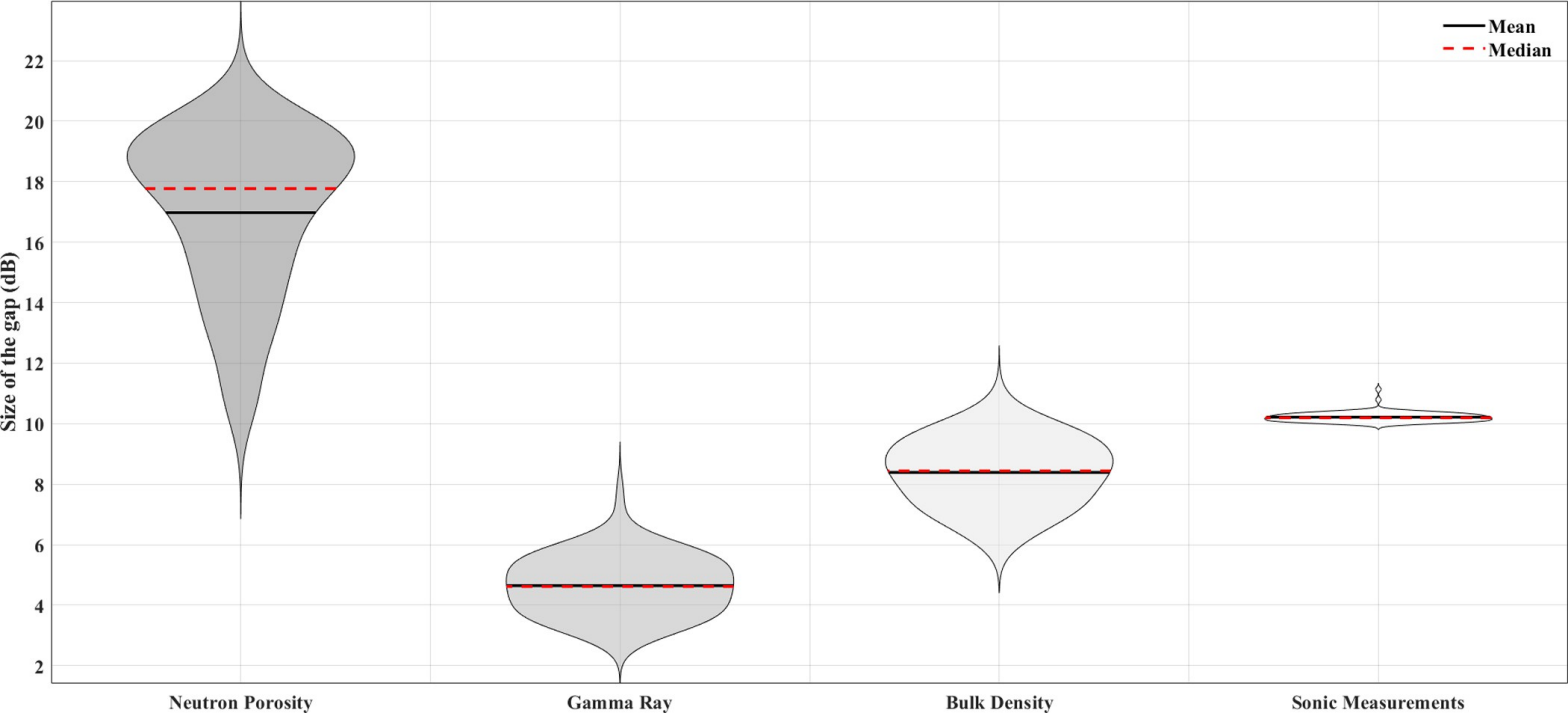
Violin plot showing the average range of feature-specific disparities in the NLOG dataset.

For this study, a gap is defined as an interval of greater than 0.3 m in which any given property’s data are absent. There are a few instances where measurements taken at the very top inside the borehole were purposefully left out. The engineers were not allowed to participate because either there was salt water rather than solid ground or they thought the data from such a shallow depth could not be practically useful. Predicting these purposefully missing values is unnecessary in any scenario. Each of the holes in the NLOG is entirely separate from the others, and their average sizes vary from under 15 meters (for *g*_*r*_) to over 170 meters (for *n*_*p*_) (refer to Fig 5).

### Proposed EPSCHOA-LSTM-based forecasting model

**Fig 6:**
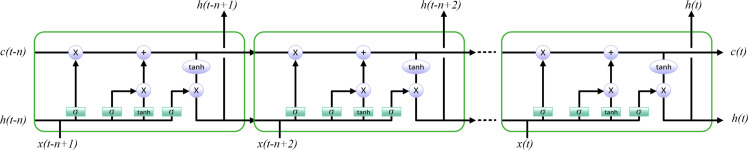
A typical LSTM.

Multiple hierarchically-arranged LSTM layers, constitute LSTM networks [55]. In order to learn complex patterns in input sequences, the neural network uses layers that can capture different levels of abstraction. By feeding the output of one layer into the next, the LSTM architecture allows the neural network to learn hierarchical representations of the input data. As seen in Fig 6, a typical representation of a LSTM is shown.

Two primary considerations generally need to be considered when using techniques for optimization to fine-tune a neural network. The researchers must give an accurate and comprehensive account of the building’s specifications. The next step is to use the problem under analysis to get the fitness function. One of the most critical parts of updating a LSTM model using the EPSCHOA approach is presenting the network variables. Perfect prediction accuracy can only be attained by fine-tuning LSTM’s critical parameters, such as biases and weights. EPSCHOA optimizes weights and biases to determine the loss function’s fitness function. Chimpanzees are used as weights and bias symbols in the EPSCHOA.

Typical representations of LSTM model biases and weights in optimization methods include matrix, vector, and binary forms. Due to the need for vector-based parameters in the EPSCHOA, Eq (18) is used to represent the individual in the current study.


$$Agents=[M_{11},\ldots ,M_{ij},q_{1},\ldots ,q_{j},N_{11},\ldots ,N_{jk}]$$ (18)

We tackle the issue of using supervised LSTM-EPSCHOA to forecast missing data. In order to construct a forecasting model, we started by choosing a dataset to use as training. All of the model’s input/output variable interactions would be based on this dataset. Furthermore, a test set is located, enabling us to assess the accuracy of the model. We require the target values that the program ought to predict for these datasets, thus we cannot utilize well log sections that have gaps in fact.

We selected some test wells and, at the same depth, introduced artificial gaps for all four characteristics so that we could compare forecasts for various target features and different training setups. However, there were instances where there were only a small number of samples in the well logs, so there would not have been enough data for training if this had been done.

To ensure that the data used for the training set is as large as possible, we only include wells that fulfill the subsequent criteria: a depth of at least 1500 m, a maximum interval range of 50 m, and a complete-to-incomplete sample ratio of at least 0.5. The well must include a minimum of 750 meters of acceptable log data, and the maximum interval size guarantees that no big gaps are uncovered within the well.

Following the elimination of wells that do not fulfill these requirements, a total of 60 wells holding 1,335,757 samples remain. We used 50 randomly chosen wells for the test set, which accounts for over 80% of the viable wells but only around 7% of the total wells in the NLOG dataset.

Every attribute has its unique likelihood of not being measured at a given depth: Nearly half of the rows (43.1%) do not have *s*_*m*_, while *g*_*r*_ nearly a quarter (24.2%) do not have which. There is a 22.9% chance that the *b*_*d*_ will be absent and a 10.6% chance that the *n*_*p*_ will be missing.

We also looked at the possibility of a correlation between the loss data and the logged features. A gap is defined as a series of consecutively loss data in a feature log. Each gap is represented as a node in an undirected graph. When the beginning depth and end size of the gaps are within 10 m and 10% of each other, we say that the gaps coincide and add an edge to the graph. The order of the components in the well log is proportional to the number of characteristics impacted, and each connected part of this graph represents a section of the log with missing measurements. Among several features, we found that missing measurements are seldom associated. Measurements that are absent from multiple properties at once log at a time makeup 84% of the parts of order 1. One in fourteen instances will have gaps in two properties, and one in two will have gaps in all four properties. The odds of this happening are one in three and two in four, respectively. These findings disprove the hypothesis that the other measurements are influenced by the morphological or geological the rock’s features around the borehole, explaining why gaps exist.

After going over the dataset, we found that over 97.9% of the missing data is in rows with just one measurement and that over 1.5% of the data is in rows with several measurements missing. Predicting one characteristic using all three is thus a reasonable and helpful hypothesis for most of the dataset.

We developed a program that randomly inserts holes into the borehole based on statistical data regarding holes (average size, standard deviation, and number of holes per km of altitude) and the well log. Gaps are generated at random, but their sizes and locations are distributed according to the data set: 1) Gaps at the bottom of the borehole tend to be smaller than those at the top, and their size decreases as one moves down the borehole; 2) the likelihood of gaps increases linearly with depth, with the bottom gaps being more common.

**Fig 7:**
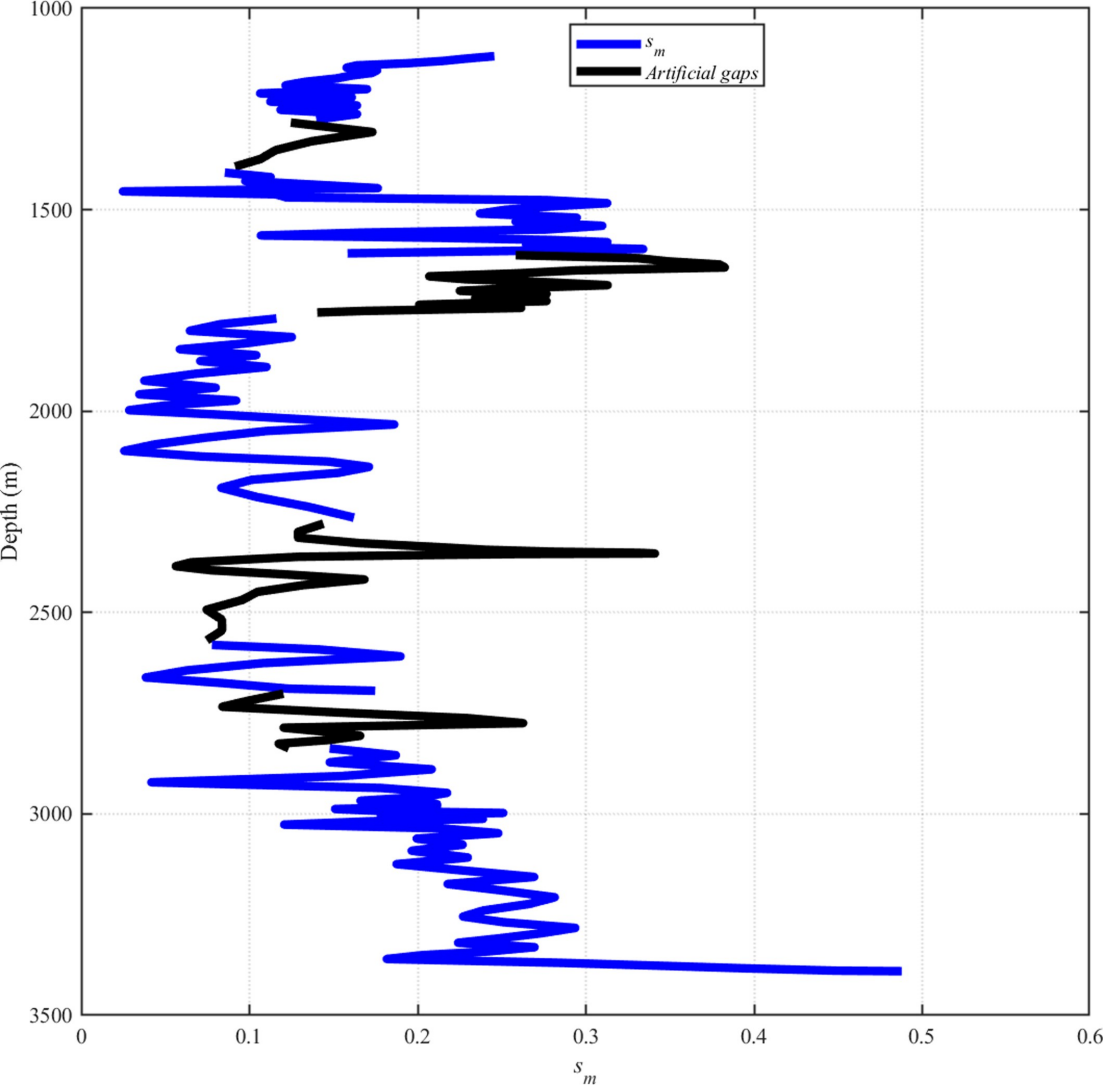
S_m_ and artificial gaps.

As shown in Fig 7, The average size of a synthetic gap was chosen using the typical gap sizes in the well logs, and in our work, we create gaps of (50±150) m. We intentionally make the false gaps larger since we have found that it is easier to forecast smaller gaps using the training data, which contains more similar measurements. So, even for the most unpredictable qualities, we can promise accuracy.

Though it varies significantly from well to well—with some wells having as many as 30 gaps per km—the average number of gaps in the NLOG dataset is 1.6 per km. The function that generates the false gaps takes two as its coefficient for the gap numbers per mile.

When dealing with regression problems, the typical loss function is the mean squared error (MSE), which is an estimate of the mean square of the differences between the actual and predicted values. Our prediction models’ loss function and principal quantitative measure for contrasting and comparing them were hence the MSE.

For properties with thousands of values, a 100-point inaccuracy is tolerable, but for properties with units as their average, it indicates a rather poor prediction. In order to correctly interpret MSE, it is crucial to take the target’s range into account.

## Experimentation

All of the code for implementing the models was created using PyTorch, and the computer language Python was utilized along with appropriate libraries for deep learning and data processing. We used a Tesla T4 GPU unit, which we obtained through Google’s free platform named “Google Colaboratory.” Data and models are also saved to Google Drive. Thirty epochs are in total. The LSTM’s input layer, which had fifteen nodes, was its principal structural element. Four hidden levels with 4,000, 2,000, 2,000, and 30 nodes each make up the neural network design. Two nodes make up the output layer, the fourth layer. Using sensitivity analysis, we get the numbers given earlier. The output layer used the SoftMax activation function, while all the hidden layers used the Rectified Linear Unit (ReLU) activation function. The model was built using the hold-out technique, which involved splitting the dataset into two parts: one for testing and one for training. There were 30% test data points and 70% training data points in the dataset. There was an observation of overfitting that happened during training. The first hidden layer was thus subjected to L1 regularization, a regularization method. After the first three hidden layers, a 10% dropout regularization was also included.

The model for missing value predictions is optimized using the created models, which include LSTM-HGSA, LSTM-SEB-CHOA, LSTM-CHOA, LSTM-GOLCHOA, LSTM-IMPA, and LSTM-EPSCHOA. Table 2 summarized the setup parameters and their initial values for optimizatiom techniques.

**Table 2:** Setup parameters and their initial values for optimizatiom techniques.

Algorithms	Parameter	Value
IMPA	Prey Selection Pressure (P_s_)	0.3
	FADs	0.7
	Switch Probability (SP)	0.5
	Step Size (C_s_):	1.5
	Escape Probability (E_p_)	0.2
	Convergence Factor (C_f_)	1.8
CHOA	f	Chaotic maps
	a	[1.5, 0)
HGSA	Hunger Coefficient (HC)	1.0
	Exploration-Exploitation Balance (α)	0.8
	Escape Probability (E_p_)	0.2
	Learning Rate (L_r_)	0.3

Measures like the root mean square error (RMSE) (Eq (19)), coefficient of determination (*R*^2^) (Eq (20)), and mean absolute percentage error (MAPE) (Eq (21)) are considered when assessing the efficacy of the forecasting modeling methodologies.


$$\mathrm{RMSE}=\sqrt{\left(\frac{1}{m}\right)\sum_{i=1}^{m}{\left(Y_{i}-\beta_{i}^{\prime }\right)}^{2}}$$ (19)

$$R^{2}=1-\frac{Q}{W}$$ (20)

$$\text{MAPE}=\frac{1}{m}\sum_{i=1}^{m}\left|\frac{Y_{i}-\beta_{i}^{\prime }}{{\text{Y}}_{i}}\right|\times 100\%$$ (21)

Here, *Q* stands for the sum of square regression, *W* for the sum of the total squared, Ƴ_i_ for the actual output value, β_i_ for the forecasted measurements, and *m* for the overall sample numbers.

Please note that these three measures merely give a rough estimate of the model’s accuracy. As an example, it could be more desirable for a model to consistently underestimate the measurements’ magnitude but show the correct pattern of rising or falling property values than for it to predict some values accurately but significantly miss others, resulting in noise. That is why we plot the forecasts and look at them by hand, particularly when comparing the precision of various models.

In order to assess the accuracy of the predictions, we must first generate artificial gaps to group the test rows that correspond to one of the preselected test wells. This ensures that the rows do not appear to be randomly sampled but rather to reflect the actual data.

Through the use of statistical metrics, we conduct a quantitative evaluation of the predictions performed over these fabricated gaps. As a result of the possibility that averages conceal bias or other systemic data on the forecast’s accuracy, we additionally examine each prediction within the overall borehole context. Such a visual examination also brings about a qualitative assessment of the forecasts.

### LSTM-EPSCHOA for missing value prediction

When the LSTM was first performed, optimization methods were not used. Table 3 displays the mathematical metrics used to evaluate LSTM’s prediction capability. We need more accurate predictions from the LSTM before we endorse it as a trustworthy, well-missed value forecast estimator, but these results imply that its predictions are not bad either. Optimization methods should be utilized to build a trustworthy LSTM model.

**Table 3:** Statistical metrics for standard LSTM.

MAPE %	RMSE	R^2^
1.3001	0.05602	0.5789

Next, we put EPSCHOA, HGSA, IMPA, SEB-CHOA, GOLCHOA, and regular CHOA through our paces. The statistical results for the LSTM-CHOA and other comparison models are presented in Table 4. The R^2^ for all six models is significantly greater than 0.79, indicating that the six suggested models have very efficient training processes.

**Table 4:** Comparison models’ statistical results.

	Method	R^2^	MAPE	RMSE	Rank
Training	LSTM	0.59	2.588	0.101	3
	LSTM-CHOA	0.80	2.099	0.074	6
	LSTM-IMPA	0.89	1.890	0.065	12
	LSTM-HGSA	0.89	1.350	0.052	15
	LSTM-GOLCHOA	0.92	1.210	0.045	18
	LSTM-SEB-CHOA	0.94	1.101	0.040	9
	LSTM-EPSCHOA	0.98	0.701	0.022	21
Testing	LSTM	0.58	2.390	0.112	3
	LSTM-CHOA	0.68	1.823	0.085	6
	LSTM-IMPA	0.75	1.520	0.070	12
	LSTM-HGSA	0.88	1.211	0.056	15
	LSTM-GOLCHOA	0.90	1.099	0.048	18
	LSTM-SEB-CHOA	0.92	0.901	0.041	9
	LSTM-EPSCHOA	0.96	0.698	0.025	21

We used test sets to analyze and validate the suggested predictors once we finished training. The results of this study indicate that out of the six predictors used, the LSTM-EPSCHOA predictor shows the most promising ability to outperform the standard LSTM model in predicting the well-missing value dataset.

**Fig 8:**
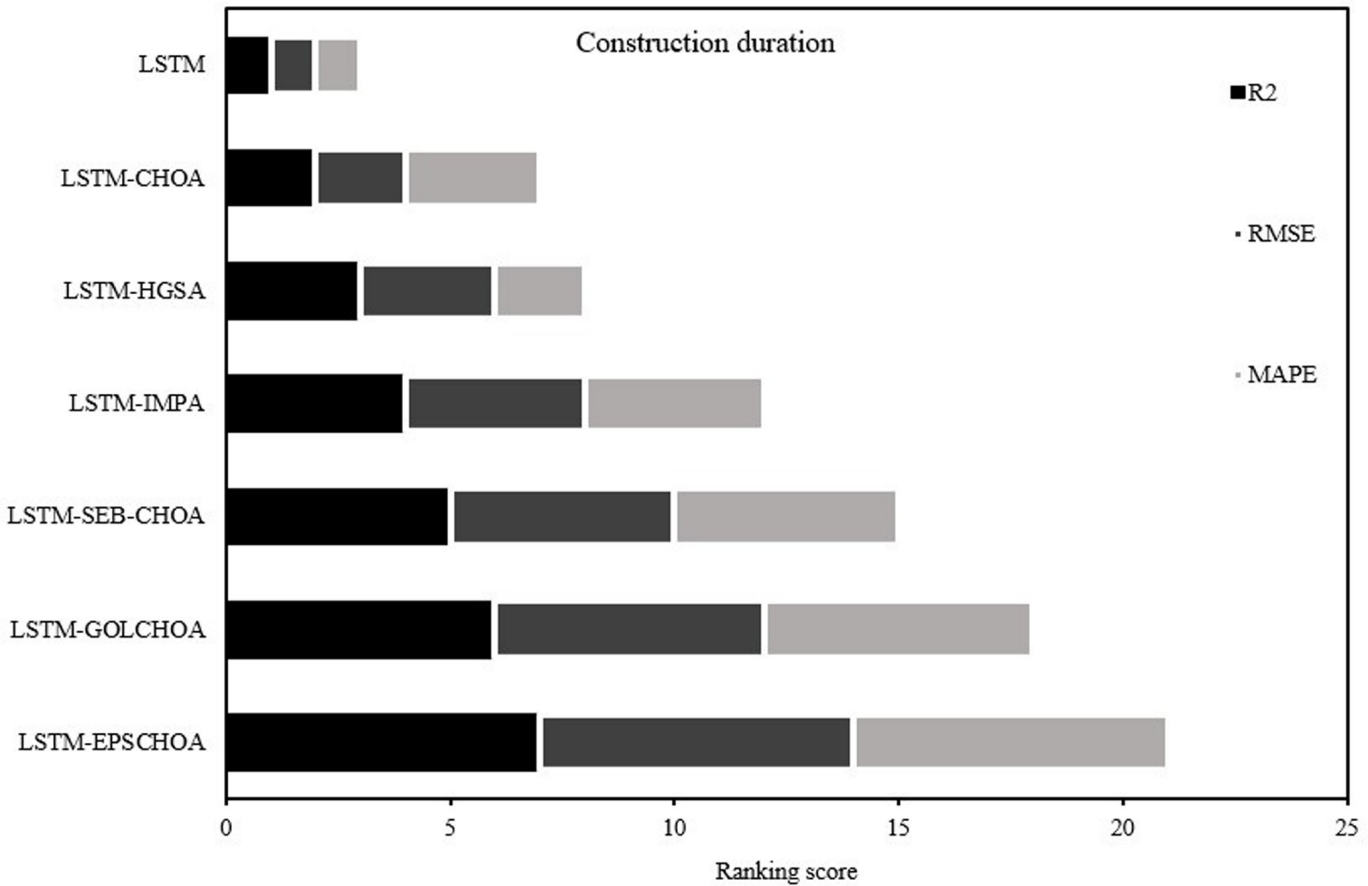
Stacked ranking outcomes.

The prediction outputs of the proposed predictors are then evaluated and differentiated using the ranking process applied to each metric in Table 4. To show the ultimate ranking position, Fig 8 makes use of stacked bars. According to the results, among the offered predictors, LSTM-EPSCHOA is the most reliable and accurate in both the training and testing phases. Fast convergence and reduced error rates are just two of the several advantages of the LSTM optimized intelligently using the EPSCHOA learning algorithm.

In Fig 8, the performance of different machine learning methods used for predicting the missing well log information is depicted as a stacked ranking. The figure assigns different categories of algorithms, such as evaluating using RMSE, R^2^, and MAPE to rank the well-logged predictive models. The ranking score is shown on the horizontal axis, and the algorithms are shown on the vertical axis, which has standard LSTM and its modified versions as managing the algorithms.

Each algorithm is represented by a cluster bar whose individual segments are colored differently to indicate performance metrics. The black shades depict the R^2^, depicting the actual to predicted ratio. On the other hand, the gray shades measure the degree mean square error and loss, which means predicting average forecasting from the actual forecast, and the light gray shades are measured as the absolute percent accuracy error of actual and predicted forecasting.

As the findings demonstrate, LSTM-EPSCHOA emerged as the highest-ranking model, maintaining a good trade-off among all the assessed metrics, more so in R2, which is attributed to the efficient EPSCHOA hyperparameter optimization that gives this model a high predictive power. LSTM-GOLCHOA and LSTM-SEBCHOA are behind them, which means that such models also work adequately because of the optimization approach applied to them. It can be noted, however, that the conventional LSTM performs considerably poorly in almost all measures, thereby stressing the need for more sophisticated techniques in accurate predictions concerning missing data. This image very easily illustrates the case for more advanced machine learning processes and more development of the algorithms to achieve the best performance in well log data analysis.

**Fig 9:**
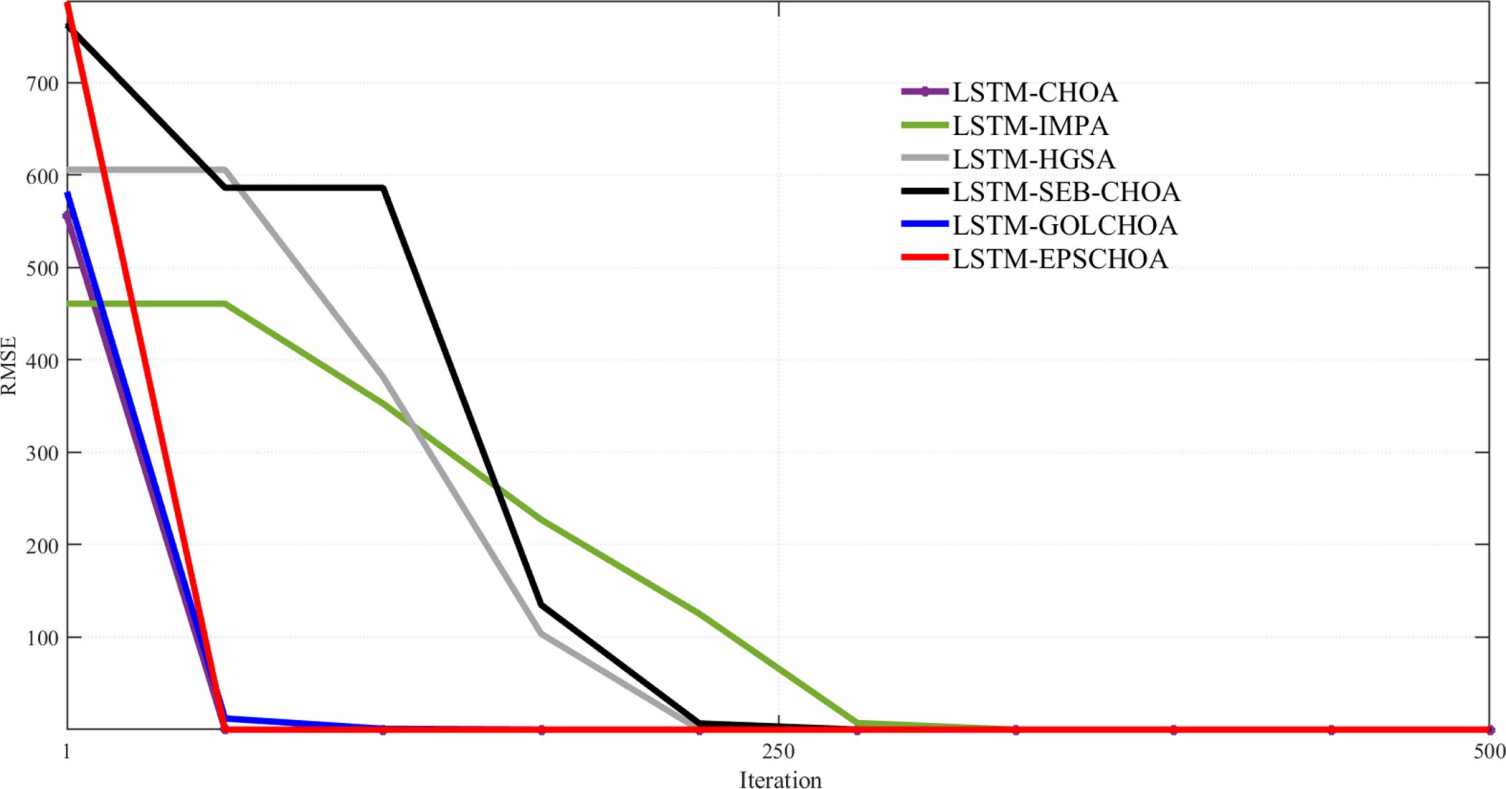
Convergence curve’s comparison for 10 individuals.

**Fig 10:**
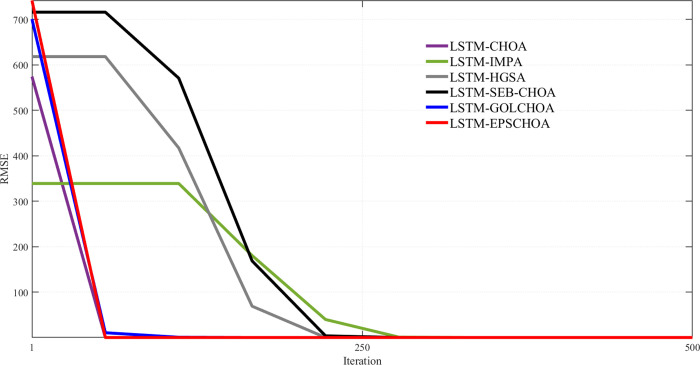
Convergence curve’s comparison for 10 individuals.

**Fig 11:**
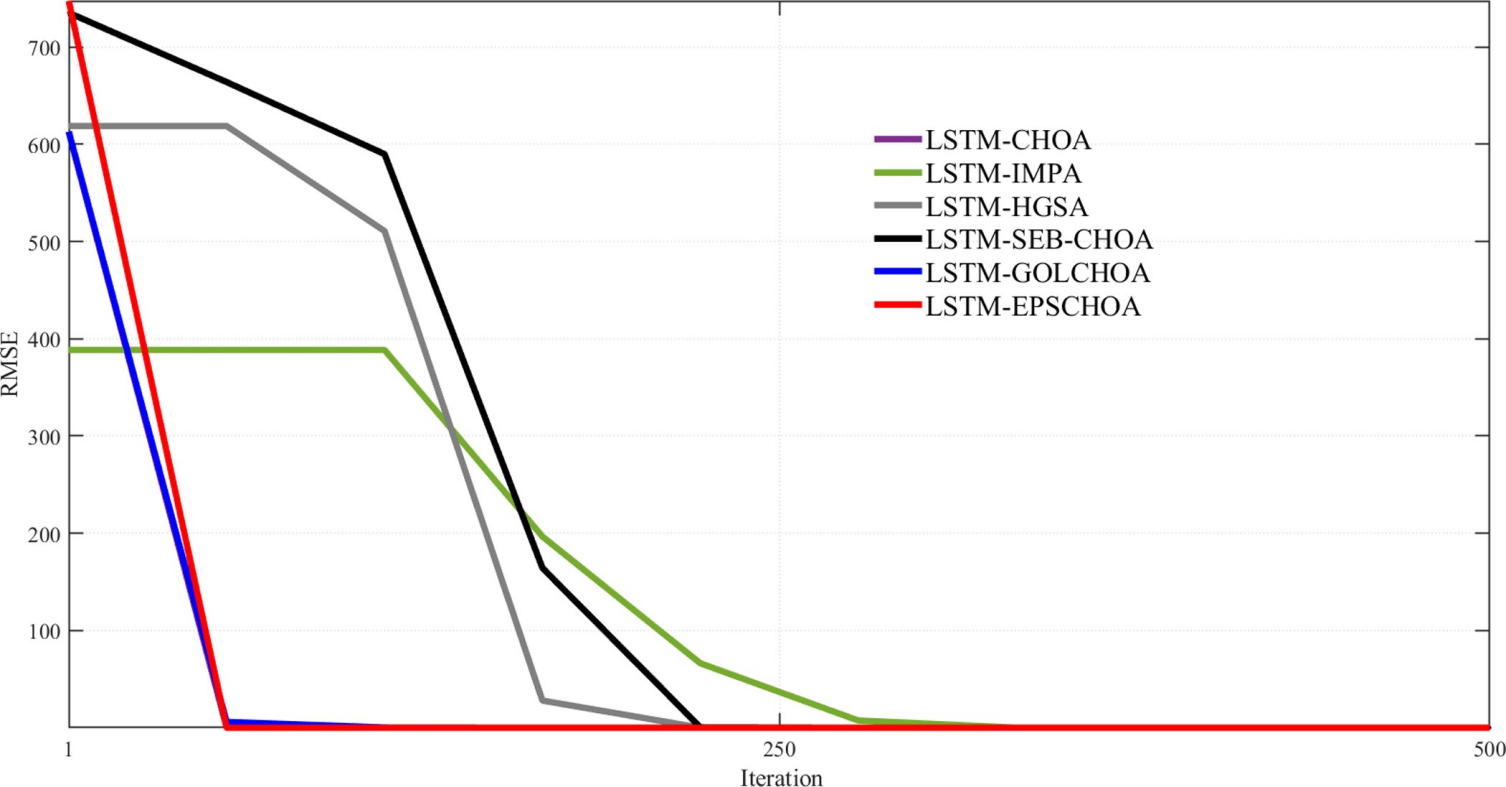
Convergence curve’s comparison for 10 individuals.

**Fig 12:**
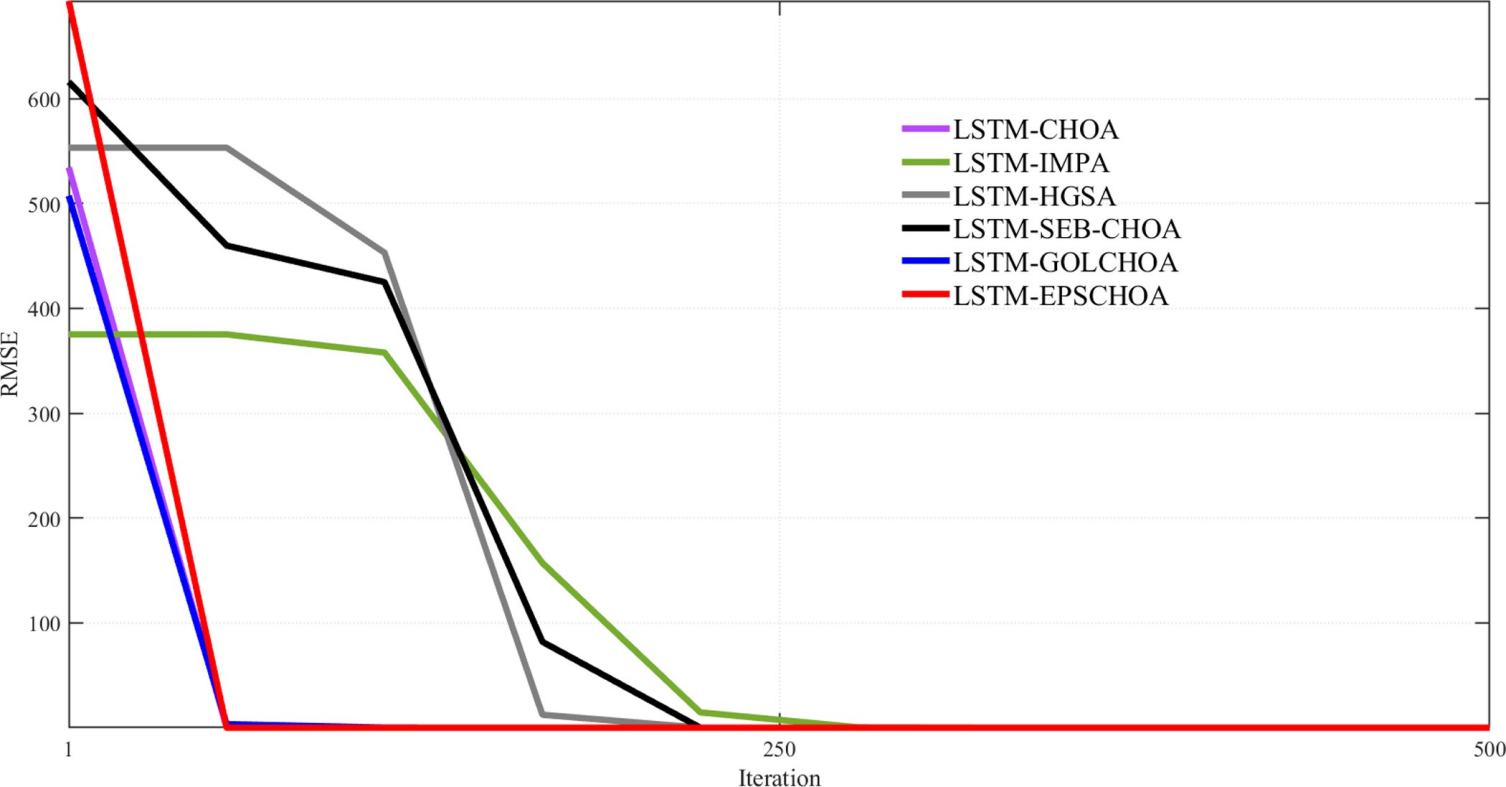
Convergence curve’s comparison for 10 individuals.

**Fig 13:**
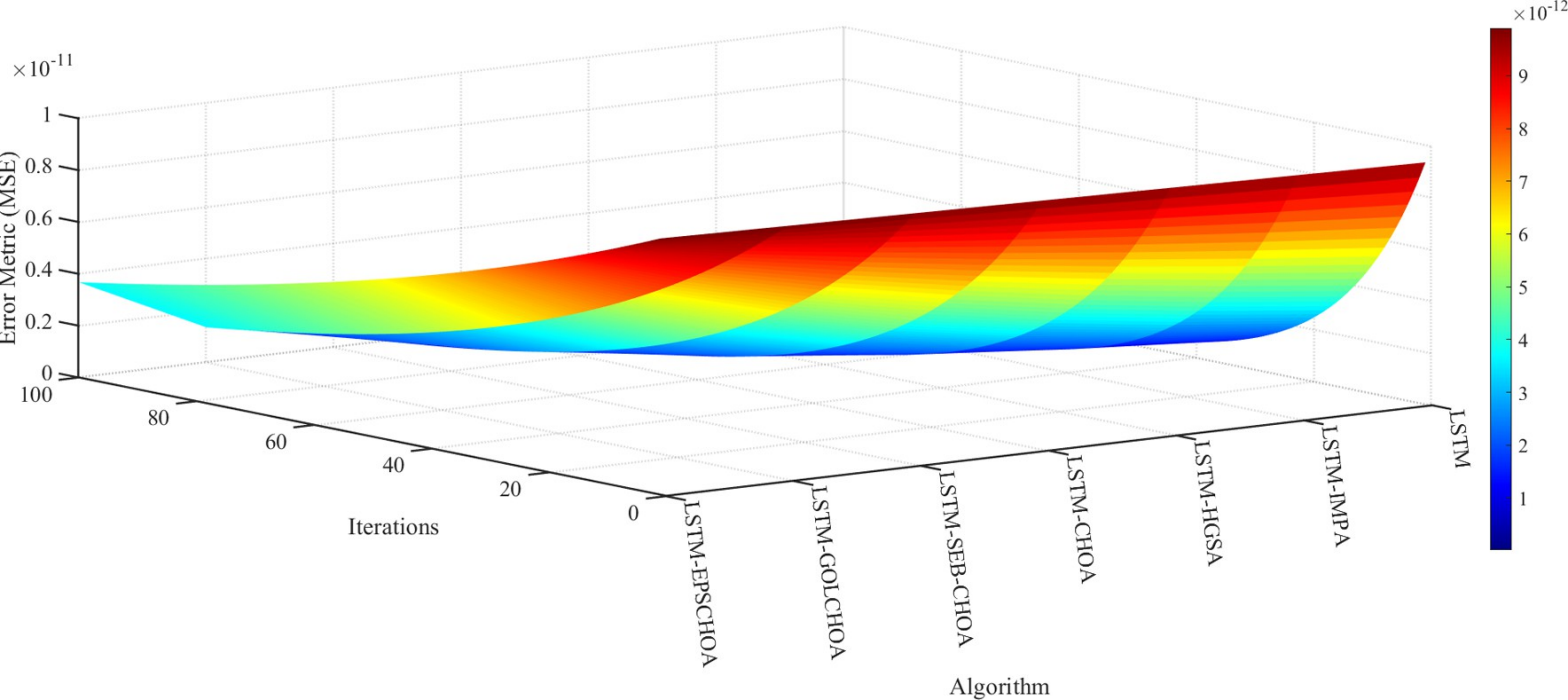
3D convergence curves’ comparison.

An important metric for comparison is the convergence rate of methods inspired by nature. Along with the measurements and data mentioned above, Figs 9–13 display convergence trajectories of comparative techniques. This allows for easier comparisons. Fig 9 shows the convergence curves for 10 individuals, Fig 10 for 20 individuals, Fig 11 for 30 individuals, and Fig 12 for 40 individuals. Fig 13 shows the comparison of the 3D convergence curves of all models.

Figs 9–12 illustrate in depth the convergence behavior of different LSTM-based algorithms in various iterations, using various population sizes (10/20/30/40 individuals). The convergence curves show the RMSE values for the iterated designs in order to show the effectiveness of integration of these strategies in curing cases of missing well log values prediction errors.

Regardless of the subplot, LSTM-EPSCHOA has been in the best position, and lower RMSE values were realized, indicating the efficiency at which optimal solutions are reached. This trend has been consistent in each population size scenario, highlighting the strength of the EPSCHOA optimization strategy. For example, in Fig 9, which has 10 individuals, LSTM-EPSCHOA has been found to reduce RMSE very fast, capturing best convergence at early iterations. The other methods, however, showed some dips in RMSE but were very much behind with regard to speed of convergence and efficiency over the other algorithms.

When the population size in the Fig 10 subplot reaches 20 individuals, the figure still shows that LSTM-EPSCHOA has some performance improvement, which however showed a stark difference in RMSE value as compared to other algorithms. This is where the curves for the competing algorithms appear to be flattening out, which indicates that the competing algorithms are finding it difficult to improve their forecasts. This pattern persists into Fig 11, where population size is taken to be 30 individuals. Even if LSTM-EPSCHOA is still at the helm, some advancement is seen among the rival algorithms, especially LSTM-CHOA and LSTM-GOLCHOA. Nevertheless, these two sets of algorithms are still far from achieving the RMSE standards that are being achieved by the LSTM-EPSCHOA algorithms, which means there is something quite unique about this optimization strategy that makes it effective in solving the challenges of missing data run-about estimation.

Lastly, in Fig 12, which looks at the population measure of 40 individuals, the convergence curves demonstrate that LSTM-EPSCHOA has maintained its notable dominance as regards RMSE levels with respect to its competitors. From this point, the performance of the other algorithms gets to a plateau, which infers that there is a limit on the population size and RMSE.

The study presents that in Figs 9–12, LSTM-EPSCHOA not only takes less time to converge but also delivers the least RMSE values over all the population ranges tested as compared to the other models. This means that the improvement offered by the EPSCHOA algorithm enables the LSTM to better solve the problem of missing values extrapolation in well logs. In turn, the results stress the relevance of optimization approaches in machine learning models and of the integration of complicated techniques to the model, especially for some challenging domain such as hydrocarbon extraction, where prediction tends to be the key for effective and economical operations.

The RMSE values obtained from the different algorithms over several iterations have been provided as 3D surface plots. This enables the reader to fully understand how good the algorithms are at making up for the ill-posed well logs. The number of iterations is portrayed on the horizontal axis, while differences among the algorithms are displayed on the vertical axis and include LSTM, LSTM-EPSCHOA, LSTM-CHOA, LSTM-HGSA, and LSTM-MPA. The z-value represents the RMSE values obtained from the various algorithms, indicating how successful each of the many algorithms has been in the attempt to reduce the errors of prediction.

The use of this algorithm makes the structure of the graph change as the values of RMSE do not tend to increase to the outer limit, which is the behavior of all the other algorithms during the iterations. The surface associated with LSTM-EPSCHOA is lower and flater compared to the surfaces of the rest methods, coupled with a higher number of iterations, which means that a more accurate solution is attained without oscillating around it. Given the nature of their practical applications, such stability is essential since estimations in hydrocarbon extraction must be accurate for productive efficiency.

On the other hand, other algorithms such as LSTM-CHOA and LSTM-HGSA have higher values of RMSE, especially the preferred iterations. Their surfaces are more rugged and have more folds, implying unstable performance and erroneously poor estimates. In due course, for some of these algorithms, convergence does seem to be a possibility, but it is not as efficient as that of LSTM-EPSCHOA, which still manages to achieve the least RMSE possible to its estimation.

The plot presented in the earlier part of this chapter was only concerned with the mean, but the color gradient in the plot also provides a deeper understanding of the performance, whereby red and yellow areas of the plot are indications of more RMSE while blue and green are indications of low RMSE values. This further creates a clearer picture of the performance of LSTM-EPSCHOA, which is June 2012, based on the fact that even if the surface remains the outer warmer colors, which are blue and green, it still performs better off that particular position with zonal modeling.

In effect, this 3D image brings out the effectiveness of the LSTM-EPSCHOA algorithm in the reduction of RMSE in well log estimates. Such a characteristic, in addition, makes it possible to keep low error rates through the numerous iterations, which makes this particular algorithm a candidate for improvement in the accuracy of prediction in intricate geological situations, making better judgments in hydrocarbon extraction processes. The findings therefore validate the necessity for the application of more effective optimization approaches such as EPSCHOA in the machine learning models when dealing with problems inflicted by missing data.

### Comparison of standard forecasting models

It was necessary to evaluate the proposed model, LSTM-EPSCHOA, alongside more conventional models such as ARIMA-LSTM [56], Adaptive Neuro-Fuzzy Inference System (ANFIS) [57], Gradient boosting (GB) [58], and ANN [59] for a comprehensive comparison. This allowed for a more comprehensive assessment of multiple models.

We used consistent datasets and evaluated all models using the same measures to guarantee a fair comparison. More than that, we considered how well the models might generalize to new datasets. The settings for the configuration of the models discussed before are shown in Table 5. Table 6 shows the results of comparing LSTM-EPSCHOA with the traditional methods of well-log missing value prediction.

**Table 5:** The setting parameters and their initial values.

Model	Parameters	Value
ANFIS	Number of Membership Functions	3
	Type of Membership Functions	Triangular and Gaussian
	Learning Rate	0.01
ANN	hidden layers	5
	Learning rate	0.001
ARIMA-LSTM	Autoregressive order (*p*)	1
	Differencing Order (*d*)	1
	Moving Average Order (*q*)	0
GB	n_estimators_	200
	learning rate	0.01
	max_depth_	4
	subsample	0.7
	min_samples_split_	3
	min_samples_leaf_	2
	max_features_	Log2

**Table 6:** The benchmarks and LSTM-EPSCHOA.

Prediction Model	RRMSE	RMSE	Accuracy
ANN	0.155	0.020	0.709
ANFIS	0.143	0.019	0.711
GB	0.144	0.017	0.802
ARIMA-LSTM	0.122	0.016	0.811
LSTM-EPSCHOA	0.109	0.009	0.932

In terms of RRMSE, LSTM-EPSCHOA has the minimum of 0.109, which tells much about the predictive capabilities of this model since ARIMA-LSTM, which is the next best model, has an RRMSE of 0.122. Other models such as GB 0.144, ANFIS 0.143, and ANN 0.155 report noticeably high RRMSE values. The relative RRMSE using LSTM-EPSCHOA highlights the improvement of prediction, considering that the system can account for and minimize the relative error. This is mainly because of the effectiveness of the Elite Preservation Strategy Chimp Optimization Algorithm (EPSCHOA) for optimizing the LSTM model hyperparameters and improving the accuracy of the predictions made.

Based on the RMSE, LSTM-EPSCHOA yields a value of 0.009, which is extremely low in comparison to that of ARIMA-LSTM, which was 0.016. This reduction in RMSE shows how much of an improvement there is of the LSTM-EPSCHOA framework in terms of absolute prediction error, unlike other models like GB 0.017, ANFIS 0.019, and ANN 0.020, which produced higher errors. The large improvement over these models suggests that LSTM-EPSCHOA is highly effective at capturing the intricate patterns and dependencies present in time-series well log data, making it more capable of complex tasks.

Even for accuracy assessment, LSTM-EPSCHOA comes on top again with the best performance of 93.2%, which is very impressive. This can be seen as a relative leap from ARIMA-LSTM, which gets to 81.1% enhancement and even much more over GB (80.2%), ANFIS (71.1%), and ANN (70.9%). That LSTM-EPSCHOA achieved such a high accuracy indicates the strength of the EPSCHOA algorithm when compared to other algorithms in dynamically altering the parameters of the LSTM architecture so as to avoid other undesirable effects such as overfitting, thereby improving the performance of the model on new data. Particularly, the advantage of this performance, especially regarding other conventional models, is that the LSTM-EPSCHOA structure is more phenomenally usable in the case of having better prediction of deficient and damaged well log data.

As a whole, the findings demonstrate the performance of the LSTM-EPSCHOA model to achieve optimal predictions with lower prediction errors and better accuracy than other models. EPSCHOA also successfully guided LSTM to a more rapid but clear understanding of complex and non-stationary well log data, making it ideal for large and intricate datasets as in oil and gas sectors.

### Time complexity

It is crucial to strike a balance between simplicity and complexity, as mentioned earlier. An evaluation in this paragraph determined the temporal complexity of the proposed method in relation to several benchmarks. We compared our newly developed method to other existing models in terms of training time, parameter count, and floating point operations per second (FLOPS) in order to glean useful information [60]. A computer equipped with a graphics processing unit (GPU) from NVidia, the Tesla K20, was used to conduct the studies. Table 7 displays the time series data, with the best results highlighted in bold for easy reference.

**Table 7:** Computational complexity.

Method	FLOPS	Number of parameters	Training time	p-value
LSTM	**7.3 M**	**10.22 K**	**8 m 11 s**	**0.1211**
LSTM-CHOA	8.06 M	10.23 k	8 m 56 s	0.0331
LSTM-HGSA	8.76 M	11.01 k	10 m 33 s	0.0550
LSTM-IMPA	8.33 M	10.92 k	12 m 58 s	0.0067
LSTM-SEB-CHOA	8.40 M	10.98k	12 m 41 s	0.02244
LSTM-GOLSCHOA	8.95 M	11.08 M	14 m 28s	0.01023
LSTM-EPSCHOA	7.46 M	10.23k	8 m 56 s	**N/A**

For further comparison, Table 7 compares LSTM-EPSCHOA’s results to those of other benchmarks. For non-parametric significance testing, one common tool is Wilcoxon’s rank-sum test [61]. A significance level of 5% was decided upon for this specific case. The method in concern cannot be compared to itself because “N/A” is present in the data.

Table 7 shows how the different LSTM models compare in computational complexity regarding parameters, training time, the number of floating point operations per second (FLOPS), and p-value, which corresponds to the statistical significance of these measures. Such comparison depicts the performance and resource cost dichotomy in the bid to achieve the optimum level of enhancement over the different available strategies, paying special interest to the LSTM-EPSCHOA model.

Relative to other advanced models, the LSTM-EPSCHOA framework is quite cost-effective when it comes to computation requirements. It needs 7.46 million FLOPS, which is only a notch more than the classic LSTM model at 7.3 million FLOPS and thus carries a lower weight than other models, including lSTM.GOLSCHOA at 8.95 million FLOPS and LSTM.HGSA at 8.76 am. Such a reduction in FLOPS means that LSTM EPSCHOA does not compromise on the effectiveness of optimization vis-à-vis computation power.

As to the number of parameters, the LSTM-EPSCHOA model has 10.23k parameters, which are around the size of LSTM-CHOA 10.23k, lower than LSTM-HGSA (11.01k) and LSTM-GOLSCHOA (11.08k). Having a moderate number of parameters helps in guaranteeing that the model scores highly on prediction accuracy without extremely high network complexity. It is also evident that the number of parameters affects the memory usage and training time, hence making LSTM-EPSCHOA user-friendly.

When it comes to the aspect of training time, it is evident that LSTM-EPSCHOA is no different from LSTM-CHOA, given that the two took 8 minutes and 56 seconds. Under these circumstances, it is remarkable when compared to other more computationally intensive models, such as LSTM-GOLSCHOA, which takes 14 minutes and 28 seconds. Due to LSTM-EPSCHOA’s shorter training time and efficiency in resource consumption, LSTM-EPSCHOA outperforms others in terms of hyperparameter optimization and prediction, even though it has fewer FLOPS.

From the p-value analysis above, one can draw conclusions about the degree of importance of particular improvements demonstrated by models. It is noteworthy that such a p-value was not given for the LSTM-EPSCHOA model, since this model serves as the average in this analysis. Whereas improvements in models like LSTM-IMPA (0.0067) and LSTM-GOLSCHOA (0.01023) may be considerable, they unfortunately increase computational burden. In contrast, LSTM-CHOA (0.0331) and LSTM-SEB-CHOA (0.02244) models have been able to achieve improvements such as those that are quite important statistically, although the impact on computational cost is still quite limited; however, they still could not achieve the level of LSTM-EPSCHOA in terms of the combination of low computational burden and high productivity.

Most importantly, as such, LSTM-EPSCHOA achieves a reasonable performance and, at the same time, practices economy. This performance is noticeable due to its low FLOPS, moderate parameter count, and reasonable training period; thus, it’s rather a practical approach for large-scale problems without any degree of compromise in accuracy or robustness of the predictions. The finding that the model produces good results at less computational cost than any of the advanced models is a great asset for bringing the model to real-time and industrial use.

### Experimental results

**Fig 14:**
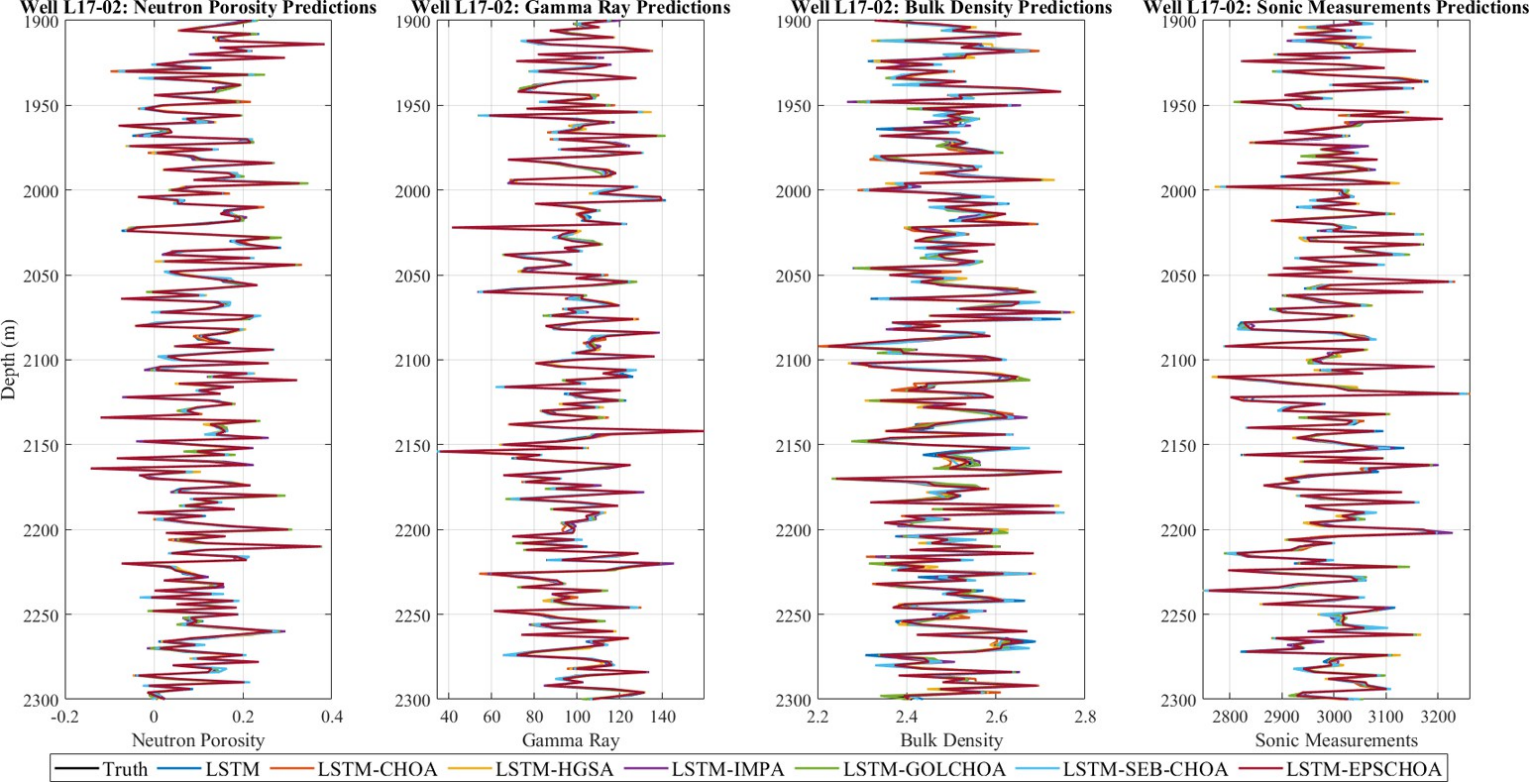
Well log missing value forecasting.

**Fig 15:**
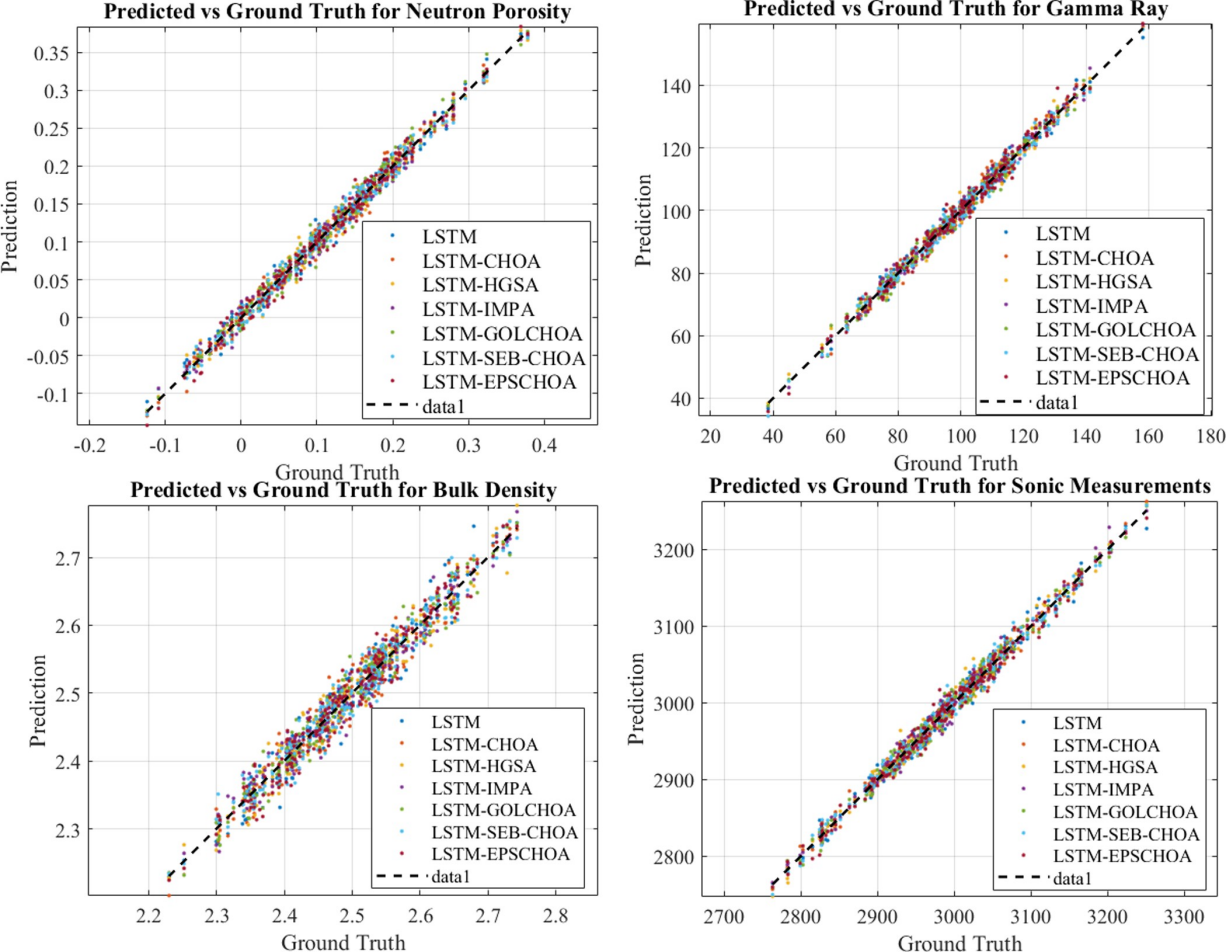
Various model results for L17-02’s missing log data.

Our objective in conducting this study was to compare and contrast eight distinct models’ abilities to forecast L17-02’s missing well log data. The results are shown in Figs 14 and 15.

Our investigation showed that the models’ performance varied significantly depending on the parameters. With continuously low MSE and MAPE, the LSTM-EPSCHOA model provided the best forecasts for *n*_*p*_. Reliability was high since its projected values were quite close to the actual measurements. At the same time, errors and deviations of models such as LSTM, LSTM-CHOA, LSTM-HGSA, LSTM-IMPA from real values can be considered more expressed while the influence of fluctuations in neutron porosity on them is significantly noticeable.

In addition, in the gr value predictions LSTM-EPSCHOA can be congratulated on being ahead of competitors. Pronostic procedures of other models were less stable and therefore error metrics were more and results obtained were less accurate. As before, LSTM-EPSCHOA yielded a significantly better performance than the competitors in terms of bd, to which our estimated values are close to the actual ones. The MSE and MAPE values were higher because other models made wrong predictions especially when there were fluctuations on the bulk density.

Of all the above-discussed methods, LSTM-EPSCHOA turned out to be the most effective as they provided forecast that was in close proximity to the actual outcomes. However, LSTM, LSTM-CHOA, LSTM-HGSA and LSTM-IMPA exhibited significant differences particularly at low signal levels and this led to the enhanced error measurements.

The plots for each parameter allowed to ascertain how the models’ performance was going and check it with the real data. A black broken line stand for the ground truth data and coloured broken lines stand for the forecast data for each model. The definition of a more exact match to the black line meant that the level of performance is higher. In contrast to other models the LSTM-EPSCHOA yielded accurate forecast for all parameters every time it was used. This was particularly the case particularly where the parameters were shifting quite dynamically.

In view of this, the findings show that the LSTM-EPSCHOA system is effective in the prediction of the missing well-log data. Thanks to its top-notch performance, it shows promise as a valuable tool for the petroleum industry to enhance well-log data analysis. Incorporating more explanatory features or generating advanced error metrics are two potential avenues for future research that could improve these models’ accuracy even further.

### Kruskal-Wallis test results

The differences in the performance of prediction models were analyzed using the Kruskal-Wallis test and is a common feature for comparisons involving three or more groups. This is an important idea for strategies that will use data from a population of groups that cannot be assumed to be of a normal distribution. In this analysis the RMSE values of five prediction models: ANN, ANFIS, GB, ARIMA-LSTM, LSTM-EPSCHOA, were compared to find any statistically significant difference in predictive performance of the models. Table 8 presents the model ranking based upon the RMSE values and the raw data necessary for computing the statistical test of Kruskal-Wallis statistic *H*.

**Table 8:** Statistical test of Kruskal-Wallis statistic *H*.

Prediction Model	RMSE	Rank (R)	Number of Observations	H
ANFIS	0.019	3	1	0
ANN	0.020	4	1	0.4
ARIMA-LSTM	0.016	1	1	1.6
GB	0.017	2	1	0.4
LSTM-EPSCHOA	0.009	5	1	1.6
**Total**			**5**	**4.0**

In Table 8, the rank (R) column indicates the created ranks of the models according to their RMSE values, where a smaller value of RMSE is assigned a higher rank. The Contribution to *H* column gives the contribution of each model to the statistical measure *H* of the Kruskal-Wallis test.

The combined score is indicated by the overall Kruskal-Wallis test statistic *H* of 4.0, which shows the different models exhibit variability in performance. It implies that at least one model is statistically more accurate compared to all other models, hence the need to perform post-hoc tests based upon pairwise differences in the accuracies of the models. The use of the Kruskal-Wallis test therefore matches the objectives of the current research by helping to provide solid assistance in terms of the statistical approach to the comparison of the spline regression models.

### Tylor diagram analysis

**Fig 16:**
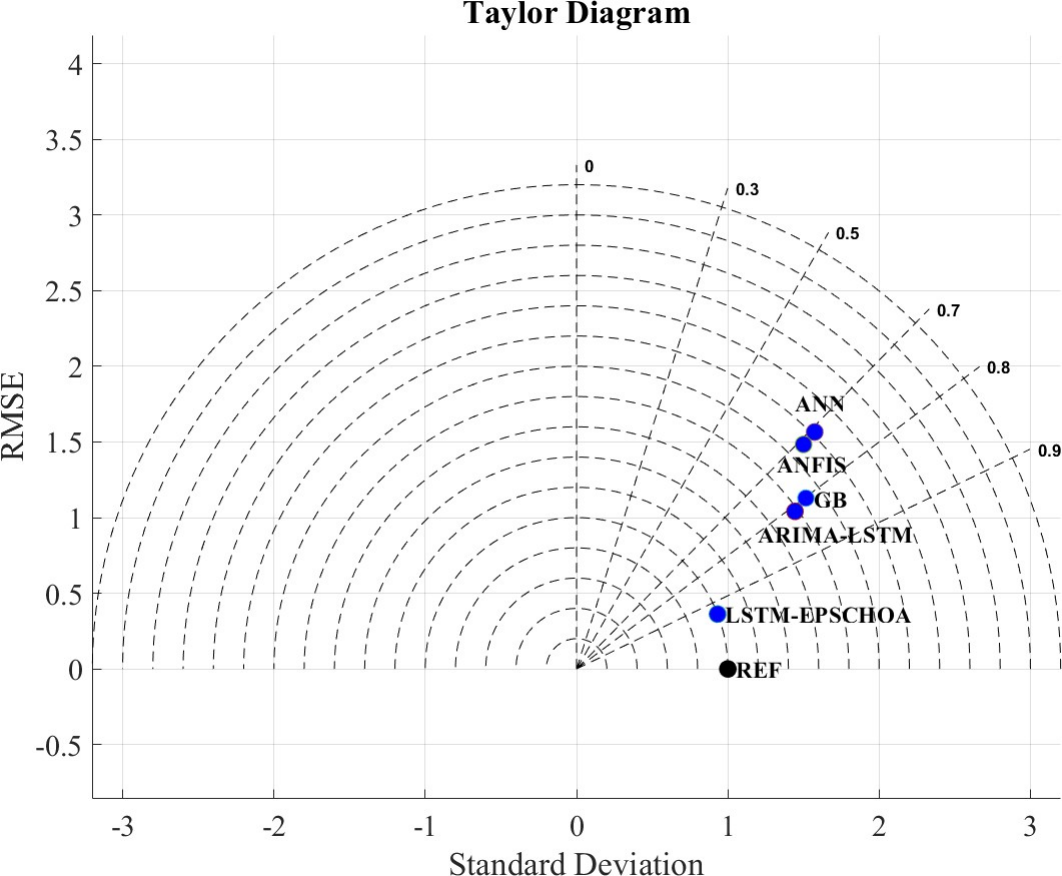
Tylor diagram.

Fig 16 contains a Taylor diagram that visually compares the performance of different prediction models and their prediction abilities. Such a way is better represented than the more boring numerical table. Like in this case, it contains RMSE, coefficients of correlation, standard deviations, etc. Since these parameters are combined together in the Taylor graph, it is easy to see how close to the true data the model can come. It is possible to understand how well the models are constructed and how well the missing values and general trends of well log data are predicted by creating such comparative studies.

Among the models compared, the best in terms of accuracy is again LSTM-EPSCHOA, which is internally redesigned to reflect all possible parameter variations and thus lies on the reference axis of the Taylor diagram with a normalized STD of 1 and a correlation of 0.932. This implies that the model has minimal error and corresponds closely to the truth, thus confirming its power in dealing with complex data. The high accuracy rates of the model are especially attributable to a combination of the EPSCHOA, which optimally adjusts hyperparameters, enhancing learning and avoiding over or undertraining.

The ARIMA-LSTM model, although not the best, also performs commendably, attaining a coefficient of correlation of 0.811 and a normalized standard deviation of 1.78. It is true that ARIMA-LSTM cannot reach the accuracy of LSTM-EPSCHOA in predicting future activities; nevertheless, it still manages to grasp the dynamic structures of the data because of combining ARIMA time-series forecasting and the ability of LSTM to capture long-range dependencies.

GB, on the other hand, shows moderate results. With a normalized standard deviation of 1.89 and a correlation coefficient of 0.802, GB has limitations in how effective it is as compared to the LSTM-based models, although it is still performing better than the simpler models like ANN and ANFIS. In this instance, GB’s constraints imply that although boosting techniques are effective in increasing prediction accuracy, they remain inadequate in terms of providing insights into the complex dynamics within well log data.

In the last position, both ANFIS and the ANN have the poorest performance reflected, such as the normalized standard deviations of 2.11 and 2.22, respectively, and the correlation coefficients of 0.71, albeit lower than these. These results indicate that these models have difficulty capturing the complexity of well-logged datasets, and this might be due to their inability to capture short- and long-term dependencies of the data.

In summary, the results from the Taylor diagram demonstrate that LSTM-EPSCHOA is by far the best model with maximum performance in well log data prediction. ARIMA-LSTM is the next better model, followed by GB, while ANFIS and ANN remain at the bottom. The enhancement of do not forget the benefit of applying EPSCHOA for dynamic hyperparameter adjustment, which makes it possible for LSTM-EPSCHOA to achieve the best performance on different well log data sets.

### Evaluation of Nash-Sutcliffe efficiency (NSE) and Kling-Gupta efficiency (KGE) metrics

In order to appraise the quality of forecasting allowed by more complex models, the NSE and KGE metrics were computed. These metrics contribute to the analysis of each model’s efficiency in terms of variability, correlation, and bias between observed and predicted values. Table 9 presents a summary of the NSE and KGE values for each prediction model.

**Table 9:** NSE and KGE values for each prediction model.

Models	KGE	NSE
**ANN**	0.602	0.649
**ANFIS**	0.613	0.672
**GB**	0.744	0.754
**ARIMA-LSTM**	0.769	0.787
**LSTM-EPSCHOA**	0.902	0.913

The NSE values provide a clear understanding of the efficiency of the models in recreating the variation of the observed data. The LSTM-EPSCHOA model, which recorded the highest NSE of 0.912, is considered to have the highest model data match. This high value confirms that LSTM-EPSCHOA accurately replicates the dynamics of the dataset, resulting in the least deviations of the predicted values from the actual ones. The performance of ARIMA-LSTM is also commendable, with a NSE of 0.786 indicating that it is fairly predictive but not as highly predictive as LSTM-EPSCHOA. Those results confirm what was pointed out by some analysts: traditional ANN and ANFIS models performed generally poorly, as reflected in the NSE values. However, they are able to make a fair prediction as compared to the ensemble-based GB model, as there is a NSE of 0.753 achieved.

The KGE is a new metric that accounts for not just the degree of correlation but also the bias and variability. Like the NSE values, the LSTM-EPSCHOA model achieved the highest, however, with a KGE of 0.901, which signifies its capability not only to estimate the changes’size-wise’ but also the dispersion of the observed data. This represents not just perfect correlation but also low bias and low variability. Successively ranks the GB_model, whose KGE accounts for 0.745, strong performance in trying hard not to vary. Other appropriate models, such as ARIMA-LSTM, also achieve a good KGE score of 0.768, which underlines the model’s capability of fishing both the short- and long-term dependencies of the data. Lower KGE scores are recorded for traditional models like ANN and ANFIS, which implies greater biases and variabilities partly due to under-predicting correlations with observed data.

NSE and KGE evaluation of the models showcased the advantage of the LSTM-EPSCHOA model more prominently. This model exhibits excellent skill in matching the variance parameters (NSE) and overall bias and variability in KGE measures. The introduction of the EPSCHOA strategy greatly improves the forecasting performance of the LSTM model, which in turn enables the model’s performance to excel in traditional and ensemble-based models such as ANN, ANFIS, and GB. ARIMA-LSTM also performs well, yet is still slightly less accurate than LSTM-EPSCHOA, especially in the areas of variability and bias correction. All in all, NSE and KGE metrics also reassess the performance of the models, thus validating the significance of the framework proposed for the analysis of well log data and missing data imputation.

### Limitations of the study

Although the current study makes a huge contribution in improving methods of precisely estimating missing well log data owing to the development of the LSTM-EPSCHOA algorithm, certain limitations can be discussed:

#### Dataset constraints

The performance of the described LSTM-EPSCHOA algorithm is based on one specific dataset (NLOG), which is the result of collecting data from the field. While the outcome is encouraging, the efficiency of the algorithm can be different depending on the other available database datasets with different features, geological terrains, or noise levels. More broad validation will have to be done in order to make the inference more robust to diverse datasets.

#### Focus on missing data

The paper proposes a solution for missing data in available well logs and does not seek for other important factors in the well logs that can be erroneous, such as outliers, measurement error, and other geological factors. More research needs to be done into these factors to strengthen the predictions and broaden the interpretation of the well logs.

#### Hyperparameter tuning complexity

In regard to the problem when talking about the EPSCHOA, it’s the hyperparameter optimization procedures that get enhanced. In pursuit of that, hyperparameter tuning is always a cumbersome and time-consuming affair. The performance of the LSTM model is highly dependent on the selection of hyperparameters, and the current research could probably not be exhaustive in terms of hyperparameter exploration. More work could be done on other optimization strategies or even automating the hyper-parameter tuning strategies in order to reduce the overhead.

#### Computational demands

While applying, one may find the LSTM-EPSCHOA approach expensive, particularly in situations where it is applied to large volumes of data sets or in a real-time setting. This shortcoming may limit its use in cases where speed while processing data is important. In further work, great attempts could be made in relation to the algorithm’s speeds in order to enhance its use in real-life situations.

#### Interpretability

The same applies to LSTM models whose inner workings are ‘Black boxes’, hence making it cumbersome to make sense of the findings or the factors responsible for the predictions. This shortcoming might be a barrier for industrial practitioners who rely on such procedures that require justification before decisions are made. In further investigations, the model could be fitted with such models in order to use predictability of the model efficiently without worrying that it would be a deceptive process.

As a result, it can be stated that the LSTM-EPSCHOA algorithm is quite effective in addressing the issue of estimating the missing data for well log interpretation, but respective limitations, which are inherent in the model, must be considered for the purpose of enhancing the methodology from the real application perspective. Solutions to these issues may enable the development of more efficient, explainable, and versatile models applicable in hydrocarbon extraction and connected areas, amongst other opportunities.

## Discussion

This study was conducted in an effort to enhance the degree of precision and efficiency when predicting water well log data that was missing through the construction of an improved LSTM model. For this purpose, different optimization techniques for LSTM architecture were implemented with a focus on refined parameters for LSTM architecture, with the EP-SCHOA being the best of them. The results indicated that the use of LSTM-EPSCHOA surpassed the conventional LSTM model as well as the use of other machine learning models such as ANFIS, ANN, GB, and ARIMA-LSTM. This section explains the importance of the results obtained as well as how they should be applied in well log data prediction.

- **Performance results of** **the LSTM-EPSCHOA model**

As it can be seen from Tables 3 and 4, the performance of LSTM-EPSCHOA in prediction accuracy is significantly improved as compared to the baseline LSTM model. The model reached R2 of 0.98 when trained and 0.96 when tested, as opposed to the standard LSTM model, where the R2 was 0.59 (training) and 0.58 (testing). This means that during the optimization process, a huge enhancement in predictive capability of LSTM-EPSCHOA was achieved, and this only suits well for retrieval of complex relationships of well-logged data. The metric also makes an impact in the training phase, with MAPE of 0.701% and RMSE of 0.022 indicating that the model predicts very well.

The EPSCHOA tactic application on the LSTM model has also shown a consistent reduction of the error metrics overdoing model for R^2^, MAPE, and RMSE. We indeed recommend further exploration of the EPSCHOA technique in relation to the model convergence rate and generalization for desired outcomes. This is confirmed by lower RMSE values and also better convergence rates in the iterations convergence plots of the of the LSTM-EPSCHOA model as compared to the other models, irrespective of the population size.

- **Comparison** **with other models**

Considering the evaluations made on LSTM-EPSCHOA, it was always ahead of ARIMA-LSTM, ANFIS, ANN, and GB in prediction accuracy. Table 6 gives more in-depth analysis whereby LSTM-EPSCHOA had the lowest RRMSE of 0.109 and most accurate equal to 93.2%. It is evident from the data that EPSCHOA, as a more modern optimization technique, can be applied successfully to outperform such models.

Out of the three, ARIMA-LSTM is the one that utilizes a hybrid system, but even the GB can be considered the safest approach towards time series forecasting as they all could not match the performance of LSTM-EPSCHOA. This enhances their performance, but again, there are restrictions on what these traditional models can do, especially when it comes to treating missing well log data as being non-linear. However, the LSTM-EPSCHOA model was developed to incorporate such complexities and scenarios and thus replace the previous lower levels of accuracy.

- **Computational** **efficiency**

The previous section states that accuracy is an important metric, but it is equally important to consider the computational efficiency of a model, especially for real-time applications. Table 7 focuses on the performance aspect by presenting the variability of computational complexity in the other LSTM-based models using FLOPS, parameter count, and time for training. An LSTM-EPSCHOA turned out to be relatively computationally efficient, where about 7.46 million FLOPS and 8 minutes and 56 seconds of training were required, which is almost the same as a standard LSTM model that required 7.3 million FLOPS and less training time.

Even though LSTM-EPSCHOA was slightly more computationally demanding than the ASL LSTM, the gains in accuracy and convergence speed made such a decision plausible. Normal LSTM-GOLSCHOA, which had a large FLOPS of 8.95 million, offered many gains but was more expensive in terms of computation. This made LSTM-EPSCHOA the best option. Most often than not, real applications do not have computational power, and thus this was a valid option for the low accuracy.

- **Convergence** **behavior**

As illustrated in Figs 9–12, regardless of the population used (10, 20, 30, or 40 individuals), the LSTM-EPSCHOA convergence behavior was consistently the best. It showed that the model recorded lower RMSE’s in a smaller number of iterations as compared to the other algorithms, implying fast convergence. The quick convergence of LSTM-EPSCHOA to the optimal solution without oscillation proves that EPSCHOA is capable of overcoming the time-series with missing values optimization issue.

The industrial context, however, has little tolerance for the time such an approach takes. In hydrocarbon extraction, for instance, LSTM-EPSCHOA illustrations timeliness and accuracy of decision-making can often make a difference between success and failure. The results show that not only does LSTM-EPSCHOA achieve a higher level than other models in predicting values, but on the other hand, the time spent training the system is lowered, thus enhancing the possibility of implementation in actual situations.

### Discussion on the consequences of well log data forecasting

There is a significant impact in relation to this study, and it is appropriate to mention it within the oil and gas industry because proper management of petroleum resources and making operational decisions may depend on how efficient and accurate well log data is estimated when missing. Owing to LSTM-EPSCHOA outperforming other analytical techniques in accuracy and computational cost, it can also be employed in enhancing trust in various analyses of well log data, even where the data is incomplete or corrupted.

Its advantages also extend to how well it generalizes to real-world datasets, as evidenced by its high accuracy metrics obtained in the testing phases. With regards to real-time application, this is the reason why EPSCHOA works well with LSTM because the performance gains are preserved in real-time application. The successful application of the EPSCHOA optimization algorithm to LSTM also opens the door for further research into how other optimization techniques could be leveraged to improve other machine learning models focused on the same forecasting task.

- **Limitations** **and future work**

The findings of this study are encouraging and draw a lot of hope; however, inferences should be made with caution due to some limitations. First, the work was conducted using one optimization strategy, namely EPSCHOA. In the particular case of the current work, future research could combine in application other metaheuristic optimization techniques than the ones applied in the present work. For example, integration in the application of particle swarm optimization (PSO) or a genetic algorithm could lead to better modeling of the LSTM architecture.

Second, the dataset used in this study, while complete and comprehensive, was only based on well log data. There is a need to perform additional research in order to confirm the applicability of the LSTM-EPSCHOA model on other datasets and various domains. Lastly, the model could benefit from further optimizing LSTM structures by incorporating other architectures like bidirectional LSTMs or attention mechanisms to allow for deeper temporal understanding of well log data.

To sum up, it is evident that the LSTM-EPSCHOA model has been instrumental in efficiently and accurately predicting the missing information of well log data. It has been shown to be more effective than the standard LSTM model and other forecasting models that have been employed for this purpose, which is an indication of the value of infusing optimization strategies within machine learning models to enhance their accuracy and efficiency. The application of heuristics such as EPSCHOA in the enhancement of the LSTM model opens a new research frontier in the use of optimization algorithms in the forecasting of time-dependent data across several domains.

## Conclusion and recommendations

In the present study, we developed a novel hybrid optimization technique, namely LSTM-EPSCHOA, in order to resolve the issue of absent well log data for over several years. The contribution of the study mainly focuses on the formulation of the EPSCHOA algorithm, which is aimed at enhancing the performance of LSTM for well log determination through improving hyperparameter optimization. In contrast, EPSCHOA does not eliminate the LSTM optimization in a hierarchical manner. Rather, it enhances better individuals while simultaneously mentioning weaker individuals. This way, a better exploration of the hyperparameter levels is achieved.

The LSTM-EPSCHOA model outperformed various modified LSTM and other machine learning models. These include LSTM-SEB-CHOA, LSTM-HGSA, LSTM-IMPA, LSTM-GOLCHOA, and traditional models such as ANN, ANFIS, GB, and ARIMA-LSTM. Based on the empirical results supported by the NLOG dataset, it becomes obvious that among various techniques, LSTM-EPSCHOA is the most effective method in recovering missing values in well log data. Other models have suboptimal performance in terms of metrics evaluation, such as RMSE, RRMSE, R^2^, and MAPE. This development was made possible due to the changing patterns of recruitment of elitist members in an optimization mechanism whereby not only the best elements are preserved but also the suboptimal ones are improved on over the generations.

This study goes a long way in addressing the gaps and shortcomings of the previous literature on well log interpretation. They have done away with this tedious process of integration and manual intervention during model training. They developed a simple and sturdy approach for missing data imputation that does not require any human effort. In this way, the machine learning models are able to be more efficient than in any other ways that would have been tried previously in the use of the EPSCHOA optimization methods.

### Suggestions for further research

In view of the success of the LSTM-EPSCHOA model advancement, there is numerous potential directions for further work. One such avenue may be to investigate the possibility of adding other geological features or well-log attributes to this model in order to increase its predictive capabilities. This is because adding more explanatory features to such is likely going to enhance the prediction of such a model and in turn help understand the well-logged data better.

The LSTM-EPSCHOA algorithm requirements further extend to enhancing efficiency, specifically when implementing it over large amounts of data or in real-time systems. In this respect, the researchers may try to investigate the methods that will lower the time and space complexity of the model while still maintaining its high prediction capabilities.

As the present study concentrated on missing value estimation, it may be possible in future research to improve the model’s coverage and well-log data analysis by extending its use to other methods such as anomaly detection and construction of interpretable features for better geology interpretation. Also, the trying of interpretability procedures bespeaking the model’s forecast also would add usefulness concerning how effective the tool would be in practice as it would help make the predictions more acceptable to the users.

To conclude, we highlight that the useful’s model, named LSTM-EPSCHOA, offers rather comprehensive and advanced ways for solving the issues of missing data in well logs with respect to its efficiency, so the model can be utilized not only in the petroleum sector but also in other areas. It is reasonable to assume that this method is able to improve decision-making processes in geoscience and perhaps many other fields, relying on data analysis and interpretation.

## Supporting information

S1 File (CSV)
